# Bioavailability, Ecological Risk, and Microbial Response of Rare Earth Elements in Sediments of the Remediated Yitong River: An Integrated DGT and Multi-Parameter Assessment

**DOI:** 10.3390/microorganisms13112443

**Published:** 2025-10-24

**Authors:** Yu Zhong, Chanchan Wu, Jiayi E, Yangguang Gu, Hai Chi, Xinglin Du

**Affiliations:** 1 Provincial Engineering Laboratory of Plant Genetic Improvement, College of Plant Science, Jilin University, Changchun 130062, China; zhongyu312@jlu.edu.cn (Y.Z.);; 2 South China Sea Fisheries Research Institute, Chinese Academy of Fishery Sciences, Guangzhou 510300, China; 3 Key Laboratory of Protection and Utilization of Aquatic Germplasm Resource, Liaoning Ocean and Fisheries Science Research Institute, Dalian 116023, China

**Keywords:** rare earth elements, DGT, bioavailability, ecological risk assessment, microbial community, urban river sediments

## Abstract

The expanding use of rare earth elements (REEs) in high-tech industrials has increased their environmental release, raising concerns about their ecological risks. This study employed the Diffusive Gradients in Thin Films (DGT) technique to assess REE bioavailability, spatial distribution, and ecological risks of REEs in sediments of the Yitong River, a historically polluted urban river in Changchun, China. Sediment characteristics (organic matter, pH, salinity), nutrient dynamics (N, P), and metal concentrations (Fe, Mn, As, etc.) were analyzed alongside REEs to evaluate their interactions and environmental drivers. Results revealed that REE concentrations (0.453–1.687 μg L^−1^) were dominated by light REEs (50.1%), with levels an order of magnitude lower than heavily industrialized regions. Ecological risk quotients (RQ) for individual REEs were below thresholds (RQ < 1), indicating negligible immediate risks, though spatial trends suggested urban runoff influences. Probabilistic risk assessment integrating DGT data and species sensitivity distributions (SSD) estimated a low combined toxic probability (2.26%) for REEs and nutrients. Microbial community analysis revealed correlations between specific bacterial (e.g., Clostridium, Dechloromonas) and fungal genera (e.g., Pseudeurotium) with metals and REEs, highlighting microbial sensitivity to pollutant shifts. This study provides a multidimensional framework linking REE bioavailability, sediment geochemistry, and microbial ecology, offering insights for managing REE contamination in urban riverine systems.

## 1. Introduction

Rare earth elements (REEs) comprise a group of 17 metallic elements, including the 15 lanthanides (from lanthanum [La] to lutetium [Lu]), scandium (Sc), and yttrium (Y) [1,2,3]. Despite the designation “rare”, REEs exhibit an average crustal abundance of 146.68 mg kg^−1^, which significantly exceeds that of many common metallic elements, such as gold and copper [4]. The rapid advancement of high-tech fields such as electronics, pharmaceuticals, new energy, and modern agriculture is driving expanding applications and deepening functional importance for rare earth elements exhibit [5,6,7].

Expanded global mining activities have introduced rare earth elements (REEs) into aquatic and terrestrial ecosystems through multiple pathways. In aquatic systems, sediments act as critical reservoirs for REE accumulation [6,7,8,9]. A key concern is their significant potential for secondary release. Altered aqueous environmental conditions, including pH, redox potential (Eh), and ionic strength, can mobilize sediment-associated REEs through physicochemical processes such as desorption and resuspension. These mobilized elements may subsequently enter the overlying water column, potentially inducing toxic effects on aquatic organisms [8].

The Diffusive Gradients in Thin Films (DGT) technique, an advanced passive sampling methodology, enables precise determination of transient solute concentrations, speciation, and spatial distribution patterns in environmental matrices including soils, sediments, and aquatic systems [10]. This approach provides innovative capabilities for assessing inorganic pollutant bioavailability [11,12], demonstrating distinct advantages in predicting heavy metal ecotoxicity to benthic organisms and evaluating their environmental bioavailability [7,13,14].

Changchun, the core city of Northeast China’s Old Industrial Base, has endured prolonged pressures from industrial intensity and population concentration. According to the 2012 National Key Monitoring Enterprises for Wastewater, 21 of Jilin Province’s 129 key wastewater emitters were located in Changchun. Approximately 80% of the city’s industrial effluent and domestic sewage discharges directly into the Yitong River, a 343.5 km watercourse with a 7515 km^2^ watershed that critically supplies municipal and industrial water [15]. Decades of industrialization severely degraded the river’s environmental quality. Pre-2016 monitoring documented acute deterioration in the urban reach, where dense concentrations of chemical and paper manufacturing industries correlated with Class V-substandard water quality, including severe black-odor phenomena. 2003 measurements revealed NH_3_-N (13.71 mg L^−1^) and BOD_5_ (10.11 mg L^−1^) levels consistent with this impairment [16]. Since initiating basin-wide remediation in 2016, Changchun has achieved visible ecological recovery; May 2023 satellite imagery indicated attenuated water coloration and revitalized riparian zones. Nevertheless, whether these improvements reflect substantive reductions in sediment pollution requires empirical validation.

Currently, clear and systematic assessments of rare earth element (REE) bioavailability and ecotoxicological risks in river sediments within Changchun City remain lacking. This study pioneers the application of Diffusive Gradients in Thin Films (DGT) technology integrated with multi-parameter analysis to systematically and comprehensively evaluate ecological risks posed by REEs and their mixtures in sediments of the Yitong River. Beyond investigating REE-specific ecotoxicological effects, we characterize the distribution of key sedimentary nutrients (organic matter, total nitrogen, total phosphorus) and heavy metals, analyzing their potential regulatory roles in REE migration and transformation. Furthermore, we elucidate microbial community structural and functional responses to REE bioavailability, revealing REE environmental behavior and ecological impacts from a microbial ecology perspective. By synthesizing DGT measurements, elemental dynamics (nutrients, heavy metals, REEs), and microbial diversity data, this work establishes a multidimensional framework for ecological risk assessment of REEs in complex sedimentary environments.

## 2. Materials and Methods

### 2.1. Sample Collection and Sediment Property Analysis

**Figure 1:**
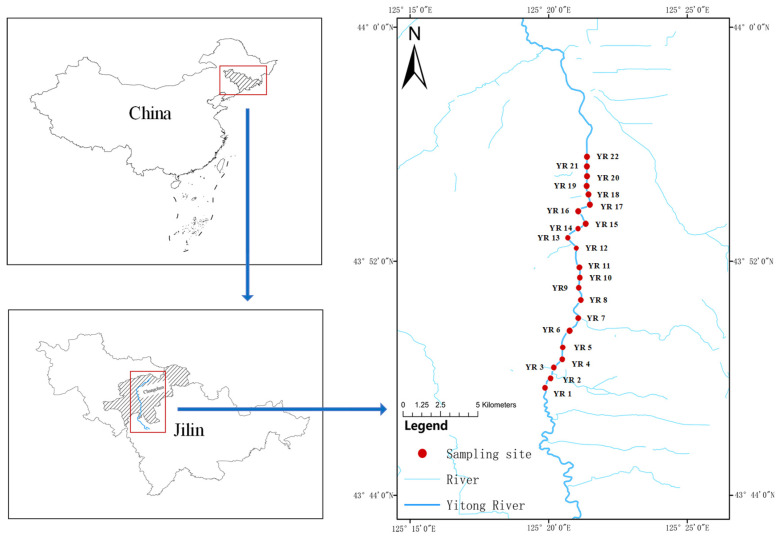
Study areas and sampling sites in China Yitong River system (YRS).

In March 2025, the research team established 22 sampling points (Figure 1) across the Yitong River basin based on geographic characteristics, pollution source distribution, and hydrological conditions. Surface sediment samples (0–5 cm depth) were collected using a Petersen grab sampler (CN-150, Ruibin Technology Co., Ltd., Guangzhou, China). For microbiological analysis, three replicate sub-samples per site were immediately placed into sterile 50 mL centrifuge tubes, flash-frozen in liquid nitrogen, and transported to the laboratory at −80 °C for DNA extraction. For geochemical analysis, samples were immediately sealed in acid-washed polyethylene bags, transported to the laboratory at 4 °C, and stored at −20 °C pending analysis. The concentration of organic matter (OM) was determined using the loss-on-ignition method. Specifically, samples were placed in a muffle furnace (CWF1100, Carbolite, Derbyshire, UK) and ignited at 550 °C to measure soil organic matter content [12]. pH and salinity were analyzed following standard analytical methods, which included sample pretreatment, instrument calibration, and simultaneous multi-parameter detection. Quality assurance was maintained through parallel sample analysis and spike recovery experiments, ensuring data comparability, reproducibility, and analytical reliability.

### 2.2. Analytical Methods

#### 2.2.1. DGT-Extraction Procedure

DGT devices (EasySensor Ltd., Ghaziabad, India) with Chelex binding gels (0.40 mm), diffusive gels (0.80 mm), and filter membranes (0.80 mm) were deployed in sediments following standardized protocols [7]. After 48 h deployment, all REEs were eluted with 2 mol L^−1^ HCl and quantified via ICP-MS (Agilent 7800, Santa Clara, CA, USA).

#### 2.2.2. REE Concentration Calculation

DGT-labile concentrations (CDGT) were derived using Fick’s first law (Equations (S1) and (S2) in Supplementary Material S1.1). Extraction efficiencies (fe) for each REE (e.g., La: 0.865, Ce: 0.872) were applied [7].

### 2.3. Toxicity Data Compilation

Acute toxicity data (LC50/EC50) for aquatic organisms (algae, crustaceans, fish, benthos) were collated from Web of Science, CNKI, and Google Scholar (Tables S1 and S2). Predicted No-Effect Concentrations (PNEC) were derived per USEPA guidelines [17].

### 2.4. Ecotoxicological Risk Models

#### 2.4.1. Single-Element Risk (RQ)

Risk quotients were calculated as [18]:

$$RQ=\frac{MC}{PNEC}$$

where *MC* refers to concentrations of rare earth elements in surface sediments. Where RQ < 1 indicates negligible risk.

#### 2.4.2. Mixture Risk (SPI Model)

The probabilistic SPI model integrated [19]: Species Sensitivity Distributions (SSD), Probabilistic Risk Assessment (PRA), Inclusion-Exclusion Principle (IEP) for joint risk (Equation (S3) in Supplementary Material S1.3.3).

### 2.5. Microbial Analysis

Genomic DNA was extracted from approximately 0.5 g of sediment (wet weight) using the E.Z.N.A.^®^ Soil DNA Kit (Omega Bio-tek, Norcross, GA, USA) according to the manufacturer’s instructions. The concentration and quality of the extracted DNA were checked using NanoDrop^®^ ND-200 spectrophotometer (Thermo Scientific Inc., Waltham, MA, USA). Amplicon sequencing of the bacterial 16S rRNA gene (V3–V4 hypervariable regions) and the fungal ITS region (ITS1–ITS2) was performed on an Illumina NextSeq2000 platform (Illumina, San Diego, CA, USA) at a commercial sequencing facility (Majorbio Bio-Pharm Technology Co., Ltd., Shanghai, China). Sequencing was performed with a 2 × 250 bp paired-end strategy, with an average sequencing depth of 60,000 reads per sample. Raw sequencing data were processed using the QIIME pipeline (version 1.91). Quality control included trimming of primers and low-quality bases (Q-score < 20) using cutadapt (v1.9.1) and DADA2 (v3.16), which also denoised the sequences and generated amplicon sequence variants (OTUs) [20]. Taxonomy was assigned to bacterial and fungal OTUs using the SILVA database (release 138.0) and the UNITE database (version 8.0), respectively. Functional prediction for bacteria was performed using PICRUSt2 (version 2.2.0) with the KEGG database (release 112), chosen for its comprehensive coverage of prokaryotic metabolic pathways and its applicability to diverse environmental samples, including sediments. Fungal functional guilds were predicted using FUNGuild (version 1.0), which categorizes fungi into trophic modes based on ecological function and is suitable for analyzing fungal communities in terrestrial and aquatic ecosystems [21].

### 2.6. Statistical Analysis

This study used SPSS v19.0 software for one-sample *t*-tests, correlation analysis (CA), K-S tests, and Q-Q plot tests. Additionally, the MASS package in R language v R4.04 software was used to calculate the overlapping regions between exposure data and toxicity data distributions.

## 3. Results

### 3.1. Sediment Characteristics

To characterize the sediments, measurements of organic matter (OM) content, pH, and salinity were performed, with results presented in Table 1. Sediment OM varied between 10.52 and 77.63 g kg^−1^, averaging 33.14 g kg^−1^. The measured pH spanned 6.51 to 9.49, with a mean of 8.07. Salinity exhibited values from 0.03 to 0.20 ppt and averaged 0.07 ppt.

### 3.2. Concentrations of Nutrient Elements, Metal Elements, and Rare Earth Elements

Pollutant concentration spectrum analysis (Table S3) revealed that DGT-labile fractions exhibited spatially stratified distribution characteristics across sampling sites. Nutrient salt components exhibited moderate-to-low concentrations: nitrate nitrogen (NO_3_^−^-N: 0.23–0.54 mg L^−1^), ammonium nitrogen (NH_4_^+^-N: 0.03–0.64 mg L^−1^), and phosphate (PO_4_^3−^-P: 0.009–0.257 mg L^−1^) (Table S3). Metal element concentrations displayed pronounced disparities, with iron (Fe: 2.35–12.38 mg L^−1^) dominating due to order-of-magnitude differences. Manganese (Mn: 39.75–5567.71 μg L^−1^) and arsenic (As: 0.53–33.11 μg L^−1^) followed at substantially lower levels, while zinc (Zn: 4.30–10.37 μg L^−1^), molybdenum (Mo: 0.87–10.67 μg L^−1^), and cobalt (Co: 0.19–4.92 μg L^−1^) demonstrated the lowest detected concentrations (Table S3). Rare earth elements were dominated by yttrium (Y: 0.203 μg L^−1^), cerium (Ce: 0.158 μg L^−1^), lanthanum (La: 0.086 μg L^−1^), and neodymium (Nd: 0.072 μg L^−1^), collectively comprising 69.40% of ΣREEs (Table S3)

### 3.3. Correlation Between Sediments, Nutrient Elements, Metal Elements and Rare Earth Elements

**Figure 2:**
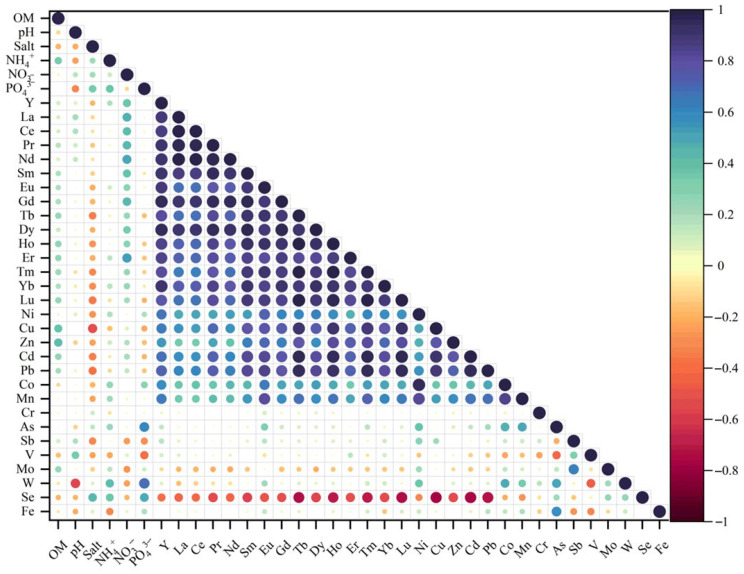
Pearson’s correlation coefficients for nutrients, heavy metal elements and REEs in surface sediments of YRS in China.

Correlation analyses were performed on sediment characteristics, nutrient salts, metal elements, and rare earth elements (REEs) within the Yitong River water body (Figure 2, Table S4). These correlations provide key evidence for elucidating the geochemical behavior and potential sources of each component. The results indicated that organic matter (OM), pH, and salinity exhibited no significant correlations with REEs. In contrast, significant covariation was observed between nutrients and metal elements. NH_4_^+^-N showed a significant positive correlation with W, while PO_4_^3−^-P correlated positively with As, W, and Se. NO_3_^−^-N displayed significant positive correlations with light REEs (LREEs: La, Ce, Pr, Nd) and medium-heavy REEs (Gd, Er). Furthermore, metals (Zn, Cd, Pb) and nutrients (PO_4_^3−^-P, NO_3_^−^-N) showed significant co-occurrence patterns.

Metal elements formed a complex and positive correlation network. The core correlation group consisted of Ni, Cu, Zn, Cd, Pb, Co, and Mn, while As, W, and Fe formed an independent covarying unit. Sb and Mo exhibited a positive correlation. Notably, elements including Cu, Zn, Cd, Pb, and Mn were generally significantly negatively correlated with Se. Furthermore, As and V, as well as V and W, exhibited significant negative correlations.

Rare earth elements (REEs) exhibited characteristic covariation patterns: Y showed significant positive correlations with La, Ce, Pr, Nd, Sm, Eu, Gd, and Tb, extending continuously across the lanthanide series to include Tm, Yb, and Lu. All REEs were significantly negatively correlated with Se.

### 3.4. Ecotoxicological Risk Assessment

#### 3.4.1. Risk Assessment of Individual Rare Earth Elements

**Figure 3:**
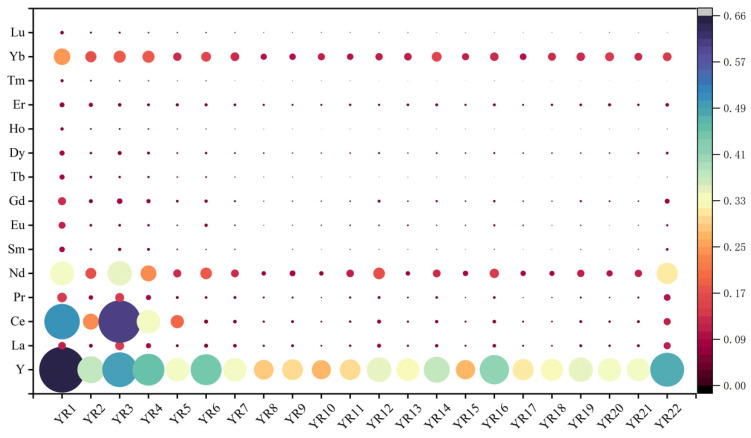
RQ of REEs at each sampling site in surficial sediments of YRS.

Based on Figure 3 and Table S5, this study systematically assessed the ecological risk quotient (RQ) of individual REEs in surface sediments of the Yitong River. Quantitative analysis revealed significant spatial heterogeneity in RQ values across 22 sampling sites for all 15 analyzed REEs (Y, La, Ce, Pr, Nd, Sm, Eu, Gd, Tb, Dy, Ho, Er, Tm, Yb, Lu), though all values remained below the risk threshold (RQ < 1), indicating an overall low-risk level. Mean RQ values ranged from 0.005 (Tm) to 0.655 (Y). Light REEs (LREEs: La [0.026–0.127], Ce [0.026–0.607], Pr [0.017–0.138]) and middle REEs (MREEs: Nd [0.063–0.349], Sm [0.005–0.079]) exhibited generally higher RQ values than heavy REEs (HREEs), exemplified by Gd [0.016–0.117]. Notably elevated RQ values for Y occurred at Site 1 (0.655) and Site 3 (0.497) (Table S5). Statistical analysis indicated high stability in REE occurrence forms, with standard deviations (SD) of RQ values typically <15% of their mean values.

Ecological risk classification confirmed negligible risk levels (RQ < 1) for all individual REEs. Elements including Eu (mean RQ: 0.010–0.100), Tb (0.006–0.075), and Tm (0.006–0.044) exhibited mean RQ values < 0.1, indicating no immediate toxicological threat to benthic organisms in the current sedimentary environment. However, spatial clustering analysis revealed 23–41% higher RQ values for Dy (0.020–0.039), Ho (0.007–0.014), and Er (0.031–0.053) at downstream sites (16–22) compared to upstream sites (1–15).

#### 3.4.2. Probabilistic Ecotoxicological Risk Assessment

**Figure 4:**
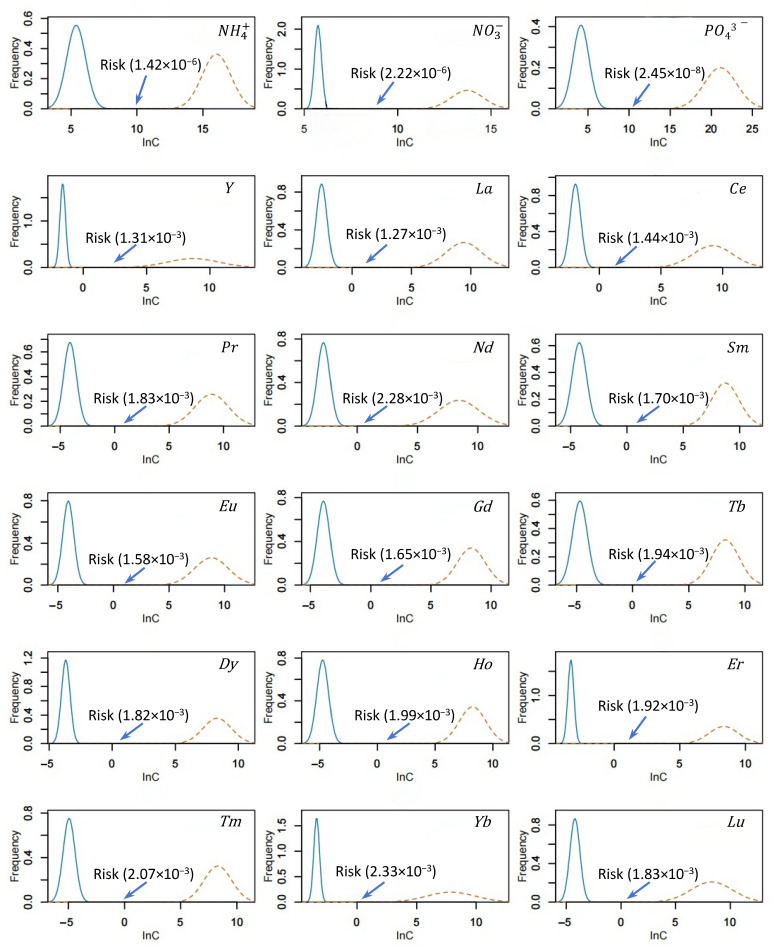
Probability density distributions of exposure and toxicity concentrations of nutrients and REEs in surficial sediments of YRS. lnC: natural logarithm-transformation concentrations. Blue solid line indicates exposure data. Yellow dot line indicates toxicity data.

This investigation employed an integrated approach combining diffusive gradients in thin films (DGT) bioavailability measurements with species sensitivity distribution (SSD) toxicity data [7] to holistically evaluate the combined ecological risks posed by nutrients and rare earth elements (REEs). Kolmogorov–Smirnov (K-S) tests verified that both the log-transformed exposure concentrations and the toxicological responses of sensitive species followed normal distributions (*p* > 0.05). Corresponding mean values, standard deviations (SD), and K-S statistics are provided in Table S6. Leveraging these parameters, normal probability distribution curves were generated for the REE exposure and toxicity datasets (Figure 4), thereby providing the probabilistic basis for subsequent risk characterization.

**Figure 5:**
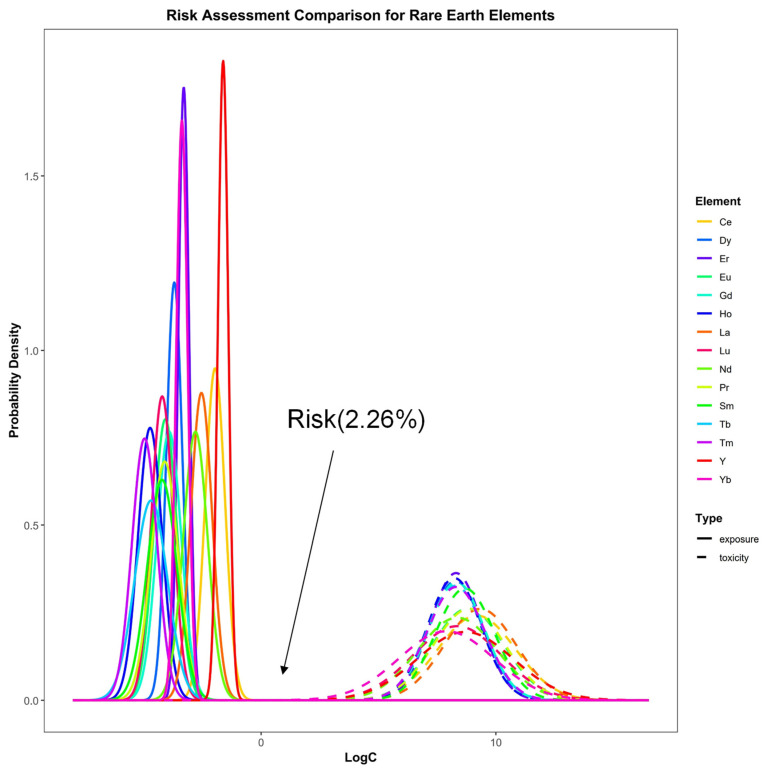
Combined risk of REEs in surface sediments of YRS. LogC: natural logarithm-transformation concentrations.

By quantifying the distributional overlap between exposure data for nutrients and individual REEs and their corresponding toxicity distributions (Figure 4), the risk probabilities for NH_4_^+^, NO_3_^−^, and PO_4_^3−^ were quantified as 1.42 × 10^−4^%, 2.22 × 10^−4^%, and 2.45 × 10^−6^%, respectively. All 15 REEs exhibited risk probabilities below 0.23%. Based on probabilistic addition [22], this study assessed the combined risk of nutrient-REE mixtures. The results indicated a 2.26% probability of combined toxic effects on aquatic organisms from surface sediments of Yitong River (Figure 5).

### 3.5. Analysis of Microbial Diversity and Its Correlation with Environmental Factors

#### 3.5.1. Analysis of Microbial Diversity

**Figure 6:**
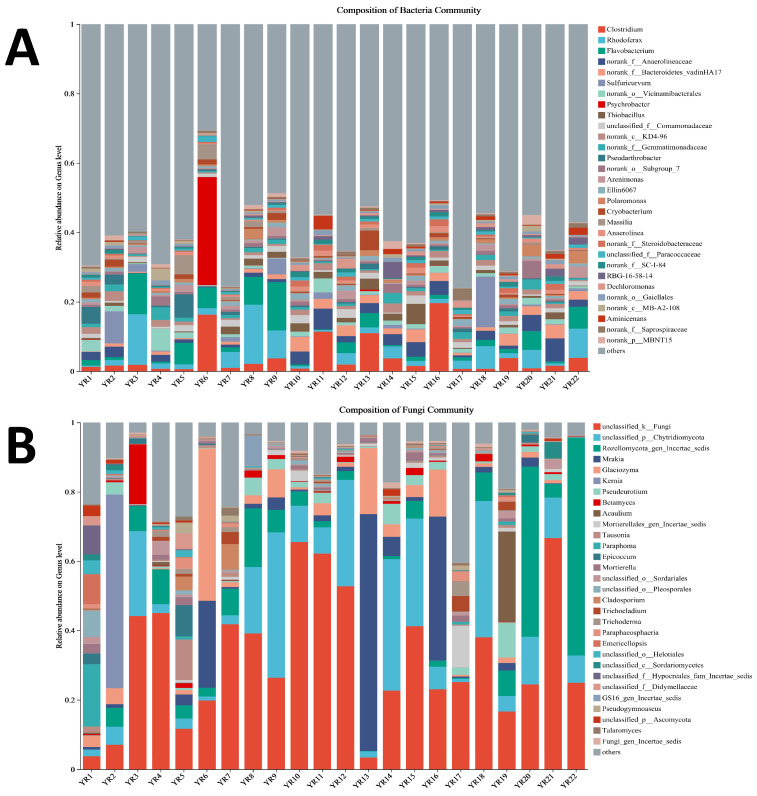
Relative abundance of top 30 genera in bacterial (**A**) and fungal (**B**) community on genus level among YRS.

The genus-level microbial community composition is presented in Figure 6. Dominant bacterial genera included *Clostridium* (relative abundance: 0.601–19.628%), Rhodoferax (0.362–17.004%), Flavobacterium (0.196–13.859%), and unclassified *Anaerolineaceae* (0.126–6.616%) (Figure 6A). Fungal dominants included unclassified Fungi (3.38–66.60%), unclassified Chytridiomycota (0.97–42.02%), *Rozellomycota incertae sedis* (undetected at YR1; max 62.89%), and Mrakia (undetected at YR4/YR22; max 68.33%) (Figure 6B).

#### 3.5.2. Correlation Analysis Between Microbial Diversity and Environmental Factors

**Figure 7:**
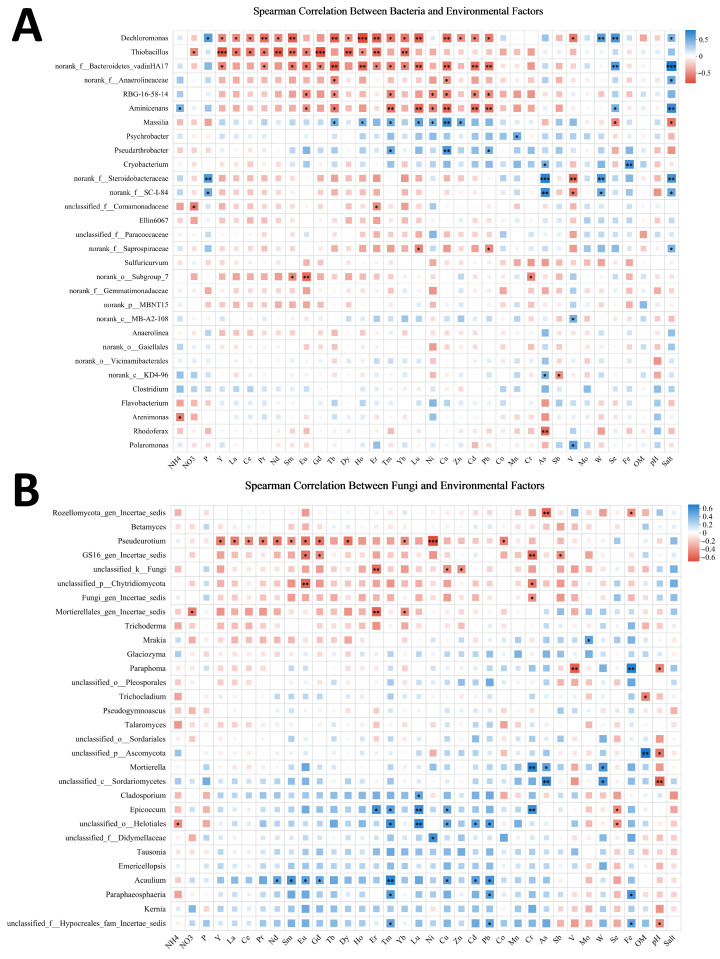
(**A**) The correlation between environmental factors and relative abundance of bacteria (genus level). (**B**) The correlation between environmental factors and relative abundance of fungi (genus level). Spearman correlation was calculated. * represents *p* < 0.05, ** represents *p* < 0.01, *** represents *p* < 0.001. (NH4 indicates NH_4,_ NO3 indicates NO_3_.

We performed correlation analysis between microbial diversity between 35 environmental factors. Figure 7 displays correlations between microbial diversity and 35 environmental factors. At the bacterial genus level, OM and pH showed no significant associations, while salinity correlated significantly with unclassified Bacteroidetes_vadinHA17, Arenimonas, and unclassified Steroidobacteraceae. Metal elements and REEs exhibited significant correlations with Dechloromonas, Thiobacillus, unclassified Bacteroidetes_vadinHA17, and Aminicenans. For fungal genera, OM correlated significantly with unclassified Ascomycota and pH with unclassified Sordariales, whereas salinity showed no significant correlations. Metal elements or REEs demonstrated significant correlations with Pseudeurotium and Acaulium.

#### 3.5.3. Microbial Function Prediction on Bacterial and Fungus Community

**Figure 8:**
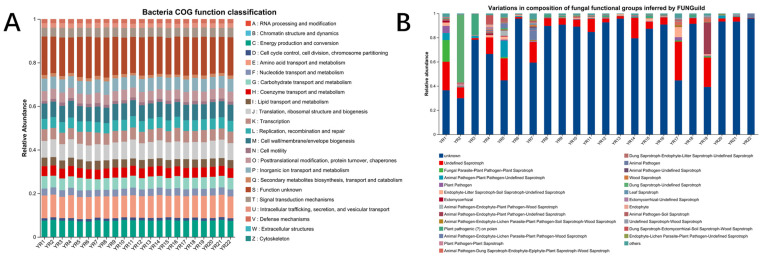
The microbial function prediction on bacterial (**A**) and fungus community (**B**) based on PICRUSt2 and FUNGuild database.

According to the microbial structure and composition, we further predicted the microbial function for each of 22 samples. First, we predicted the functional abundances on the bacterial composition in each sample based on 16S rRNA using PICRUSt2 (Figure 8A). We observed highly similar COG (Clusters of Orthologous Groups of proteins) functional profiles across all 22 samples, demonstrating no significant functional bias at the bacterial level. The composition of fungal functional groups was inferred using the FUNGuild database. We identified large variations in the composition of fungus function among 22 samples (Figure 8B). Saprotrophic fungi with varying abundances were detected in samples from the upper sampling sites of the Yitong river (sites YR1 to YR5). We also observed that the relative abundance of saprotrophic fungi exceeded 15% in sampling sites YR7, YR17, and YR19. This indicates that fungi at these sampling sites predominantly exhibited the biological function of decomposing organic matter for nutrient acquisition. In contrast, the abundance of saprotrophic fungi significantly decreases in the residential areas of the Yitong River watershed, suggesting distinct fungal ecological roles compared to other sampling sites along the river.

## 4. Discussion

Our study provides a detailed assessment of REEs in Yitong River sediments; however, some limitations should be addressed. As a case study of a single, unique river system following remediation, the findings of this study are context-specific and may not be fully representative of all river ecosystems. Furthermore, sampling was conducted in March 2025, reflecting the conditions during a single season. Seasonal variations in hydrology, temperature, and anthropogenic discharge may influence pollutant behavior. Future studies should incorporate long-term monitoring across multiple seasons and advanced data analysis techniques to better elucidate the complex interactions and temporal dynamics of REEs and their ecological risks in remediated urban rivers.

This study investigated the co-occurrence patterns of nutrient elements, metal elements, and rare earth elements (REEs) in Yitong River sediments, along with the environmental factors driving their bioavailability. As indicated in Table 1, significant spatial heterogeneity was observed in DGT-available ΣREE concentrations within surface sediments. Light rare earth elements (LREEs: La–Eu) dominated the concentration profile, followed by heavy rare earth elements (HREEs: Gd–Lu). The ΣREE levels in this study were an order of magnitude lower than those reported for Pearl River Estuary sediments (7.02–16.06 μg L^−1^), likely reflecting distinct watershed geology and heterogeneous anthropogenic impacts [7]. Correlation analyses were performed on sediment characteristics, nutrient salts, metal elements, and rare earth elements (REEs) within the Yitong River water body (Figure 2, Table S4). These correlations provide key evidence for elucidating the geochemical behavior and potential sources of each component. The results indicated that organic matter (OM), pH, and salinity exhibited no significant correlations with REEs, suggesting that REE distributions in these sediments were relatively independent of these sediment properties. In contrast, significant covariation was observed between nutrients and metal elements. This pattern suggested that nitrogen and phosphorus nutrients may act as a transport pathway for specific elements (e.g., W, As, Se, LREEs, M-HREEs) into the water body via agricultural runoff or wastewater discharge. Their spatial correlation likely reflected the combined influence of industrial wastewater discharges (a primary source of metals) and agricultural non-point source inputs (a dominant source of nutrients) within the watershed. Metal elements formed a complex and positive correlation network, suggesting that industrial pollution sources may influence element distribution through specific transport pathways or phases. Furthermore, As and V, as well as V and W, exhibited significant negative correlations. These associations likely arose from the differing behaviors of redox-sensitive elements under fluctuating environmental conditions. This inverse correlation pattern contrasted sharply with the positive covariation observed among Fe, As, and W, suggesting that reactions occurring at iron oxide interfaces may govern the mobility of these elements.

Hydrochemical monitoring, aquatic biodiversity indexes, and habitat evaluation are routinely used to assess the ecological health of rivers [23,24,25]. Hydrochemical monitoring primarily involves quantifying the concentrations and dynamic changes in nutrients (such as nitrogen and phosphorus) and pesticide contaminants in water bodies. This approach objectively assesses the physicochemical health of the watershed, identifies stress sources, and evaluates the effectiveness of management strategies. Xia et al. conducted a long-term investigation on the groundwater of Chaobai river (Beijing, China) by using the hydrochemical evolution [23]. Their findings demonstrated the dynamic changes in Ca^2+^, Na^+^, K^+^, and Cl^-^ after long-term infiltration of reclaimed water, implying that differences in hydrochemical evolution were mediated synergistically by sediment thickness and geochemical processes. While hydrochemical monitoring can be considered an “instant check-up” for a watershed, aquatic biodiversity symbolizes its “long-term record”. This represents the cumulative influence of environmental factors on water bodies over time. Simonin et al. conducted a comprehensive analysis of aquatic biodiversity indices across a wide variety of taxa and concluded that coal surface mining contributed to the extinction of 40% of river biodiversity [24]. REEs analysis, on the other hand, has become an increasingly essential tracing approach in watershed evaluation. It goes beyond conventional hydrochemical monitoring and other methods by providing unique and extremely accurate information on the sources of these elements.

In this study, we determined the concentrations of each type of REE in the Yitong River. Considering the potential harmful effects of these REEs, we used models to assess the impact of each REE present in the Yitong River on biological systems, as well as the overall adverse effects of all REEs combined. The results exhibited characteristic covariation patterns, all REEs were significantly negatively correlated with Se, indicating that REE enrichment processes may be inversely associated with the geochemical behavior of selenium. This study also developed a three-dimensional association model integrating “element concentration-sediment characteristics-bioavailability” to elucidate the distinct geochemical behavior of REEs in cold-region urban river sediments and their coupling mechanisms with conventional pollutants. The model identified homogeneous REE migration, composite metal pollution inputs, and nutrient-driven interface processes as the key mechanisms governing element distribution within the study area. Patterns of the element distribution in the Yitong River sediments reflected the combined influence of rare earth mineral weathering, industrial wastewater discharges, and agricultural non-point source pollution. These correlation characteristics provided a scientific basis for establishing basin-specific environmental benchmarks, tracing pollution sources, and improving watershed ecological risk management strategies.

Based on Figure 3 and Table S5, this study systematically assessed the ecological risk quotient (RQ) of individual REEs in surface sediments of the Yitong River, indicating an overall low-risk level. Notably elevated RQ values for Y occurred at Site 1 (0.655) and Site 3 (0.497), suggesting potential association with historical industrial activities within the watershed. However, spatial clustering analysis revealed 23–41% higher RQ values for Dy, Ho, and Er at downstream sites (16–22) compared to upstream sites (1–15), potentially reflecting urban runoff influences. In summary, RQ values for all individual REEs remained below the risk threshold (RQ < 1) at every sampling site (Figure 3), indicating limited immediate ecotoxicological risk under current conditions. However, potential cumulative/synergistic effects of REE mixtures and their long-term sediment accumulation suggest ecological risks in the study area should not be disregarded. Consequently, further research should refine risk assessment frameworks for mixture toxicity scenarios, evaluate long-term exposure effects, and finally adapt protocols to address evolving regulatory guidelines and environmental conditions.

This investigation employed an integrated approach combining diffusive gradients in thin films (DGT) bioavailability measurements with species sensitivity distribution (SSD) toxicity data [7] to holistically evaluate the combined ecological risks posed by nutrients and rare earth elements (REEs). The results indicated a 2.26% probability of combined toxic effects on aquatic organisms from surface sediments of Yitong River (Figure 5). The application of species sensitivity distributions (SSD) in probabilistic risk assessment (PRA) provides a framework for quantifying ecological risks of chemicals. Key advantages of this approach included probabilistic risk quantification, consideration of interspecies variability, empirical data reliance, methodological flexibility, and risk-based decision support. However, a significant limitation is insufficient biological toxicity data to adequately characterize community-level effects. To address this limitation, prioritizing toxicity data representative of the study area’s biological community is essential. This study mitigated data constraints by screening aquatic toxicity data from ecologically relevant species for the target water body, thereby enhancing the representativeness of risk assessments for nutrients and rare earth elements (REEs) on aquatic communities. This study also integrated DGT-derived bioavailable concentrations with aquatic toxicity data, applying species sensitivity distributions (SSD) and probability summation models to assess ecological risks from nutrients and REEs in Yitong River sediments. Results indicated a low probability (2.26%) of combined toxic effects on aquatic communities. The approach addressed toxicity data limitations through rigorous selection of ecologically representative species for the study area.

Microorganisms mediate critical ecological processes including element cycling, organic matter degradation, soil structural formation, and plant nutrient provision [26]. As primary constituents of soil biomass, bacteria and fungi exhibited high environmental sensitivity, enabling rapid response and adaptation to environmental shifts, including variations in metal and rare earth element (REE) concentrations [27]. Consequently, microbial community composition and diversity served as key bioindicators for assessing soil environmental change and pollution status [28]. This aligned with findings by Chao et al. [29]. Their findings demonstrated that soil bacterial communities in REE-contaminated sites were co-regulated by plant diversity, nutrient limitations, and REE concentrations.

In our results, we found that Dechloromonas and Pseudeurotium showed significant correlations with REEs, respectively. Previous studies have reported that taxa of the genus Dechloromonas are the prevailing hydrogenotrophic denitrifiers in nitrate-polluted aquifers, and the ability of hydrogenotrophic denitrification under the given conditions is species-specific [30]. It has also been reported that chlorinated ethanes are common groundwater contaminants in China. The unique metabolic capacities of Dechloromonas strains imply that they may play important roles in site remediation [31]. These findings suggest that the abundance of this genus can directly reflect the degree of water pollution. However, there have been no reports linking Pseudeurotium to water pollution. Therefore, the correlation observed in this study between this fungal genus and REEs in water requires further investigation to clarify the underlying reasons.

According to the microbial structure and composition, we further predicted the microbial function for each of 22 samples. We observed highly similar COG functional profiles across all 22 samples, demonstrating no significant functional bias at the bacterial level. In contrast, the abundance of saprotrophic fungi significantly decreases in the residential areas of the Yitong River watershed, suggesting distinct fungal ecological roles compared to other sampling sites along the river.

## 5. Conclusions

This integrated assessment of REEs in Yitong River sediments revealed a low but heterogeneous bioavailability of DGT-labile REEs (ΣREEs: 0.453–1.687 μg L^−1^), dominated by light REEs (50.1%). Concentrations were significantly lower than industrial estuaries due to watershed geology and anthropogenic gradients. Although single-element Risk Quotients (RQ < 1) indicated negligible acute ecological risks, probabilistic modeling identified a 2.26% combined risk probability for REE-nutrient mixtures, highlighting potential synergistic threats. Critically, REE distribution shows no correlation with sediment properties (OM, pH, salinity), instead exhibiting strong synergies with nitrogen or phosphorus nutrients and metals (As, W, Fe), confirming anthropogenic inputs (industrial effluents, agricultural runoff) as primary drivers over natural factors. Microbial communities further signal adaptive responses to pollution stress, with taxa like Thiobacillus (bacteria) and Pseudeurotium (fungi) significantly correlating with REE-metal concentrations, positioning them as sensitive bioindicators. Collectively, these findings revealed a remediation paradox: although surface water quality has improved, sediments harbor transformable REE reservoirs with secondary release potential, confirming that ecosystem recovery transcends visible restoration and demands acknowledgment of sediment-bound legacy pollution.

## Figures and Tables

**Table 1:** Mean concentrations of all rare earth element (∑ REEs), light REEs (LREEs), and heavy REEs (HREEs) (μg L^−1^) (mean ± SD). Proportions of LREEs/HREEs in surficial sediments were taken from the YRS.

Site	∑REEs		LREEs		HREE		LREEs/HREEs		OM (g·kg^−1^)		pH		Salt (ppt)	
	Mean	S.D.	Mean	S.D.	Mean	S.D.	Mean	S.D.	Mean	S.D.	Mean	S.D.	Mean	S.D.
YR 1	1.69	0.01	0.88	0.01	0.44	0.00	2.00	0.01	45.33	0.93	7.59	0.06	0.05	0.00
YR 2	0.88	0.01	0.41	0.01	0.26	0.00	1.54	0.03	49.94	0.71	7.32	0.19	0.04	0.00
YR 3	1.47	0.03	0.91	0.03	0.29	0.00	3.18	0.08	30.38	0.41	8.40	0.04	0.04	0.01
YR 4	1.04	0.00	0.55	0.00	0.24	0.00	2.33	0.02	32.41	2.04	7.98	0.08	0.03	0.01
YR 5	0.67	0.01	0.30	0.00	0.18	0.00	1.70	0.01	60.79	1.21	7.41	0.25	0.04	0.01
YR 6	0.87	0.00	0.42	0.01	0.20	0.00	2.13	0.06	21.57	1.12	8.89	0.07	0.04	0.01
YR 7	0.67	0.06	0.34	0.04	0.15	0.01	2.20	0.07	12.93	0.63	9.26	0.06	0.04	0.01
YR 8	0.47	0.01	0.20	0.00	0.12	0.00	1.75	0.02	33.04	0.22	9.37	0.02	0.04	0.02
YR 9	0.55	0.00	0.27	0.01	0.12	0.00	2.27	0.08	32.95	0.44	8.67	0.03	0.08	0.01
YR 10	0.45	0.02	0.19	0.01	0.12	0.00	1.63	0.16	23.75	2.77	9.15	0.11	0.08	0.00
YR 11	0.56	0.00	0.28	0.00	0.12	0.00	2.22	0.02	29.80	0.63	7.61	0.07	0.17	0.02
YR 12	0.74	0.00	0.40	0.01	0.14	0.00	2.88	0.11	45.60	1.25	8.14	0.02	0.06	0.00
YR 13	0.52	0.05	0.21	0.02	0.13	0.01	1.63	0.04	60.87	0.41	7.54	0.11	0.04	0.01
YR 14	0.69	0.00	0.34	0.01	0.15	0.00	2.24	0.08	77.63	0.08	8.47	0.27	0.04	0.01
YR 15	0.48	0.01	0.21	0.00	0.12	0.00	1.80	0.01	31.51	1.20	8.45	0.11	0.12	0.01
YR 16	0.74	0.01	0.36	0.01	0.15	0.00	2.38	0.11	12.89	1.23	9.30	0.05	0.08	0.01
YR 17	0.48	0.00	0.21	0.00	0.11	0.00	1.87	0.04	16.23	0.51	6.64	0.09	0.04	0.01
YR 18	0.49	0.00	0.20	0.00	0.12	0.00	1.68	0.04	15.82	1.65	6.72	0.06	0.07	0.00
YR 19	0.62	0.01	0.29	0.01	0.14	0.01	2.14	0.09	23.36	0.36	6.51	0.04	0.15	0.01
YR 20	0.56	0.01	0.24	0.01	0.14	0.00	1.68	0.07	10.52	0.50	8.02	0.04	0.05	0.01
YR 21	0.60	0.00	0.30	0.01	0.13	0.00	2.37	0.11	31.32	0.39	6.69	0.06	0.20	0.00
YR 22	1.19	0.01	0.72	0.01	0.20	0.01	3.69	0.21	30.54	1.09	9.49	0.08	0.15	0.00

## Data Availability

The original contributions presented in this study are included in the article and Supplementary Materials, further inquiries can be directed to the corresponding authors.

## Supplementary Materials

The following supporting information can be downloaded at: https://www.mdpi.com/article/10.3390/microorganisms13112443/s1, Text S1: Experimental Methods; Table S1: REE-acute-toxicity data and the corresponding values of Predicted No Effect Concentration (PNEC); Table S2: Acute Toxicity Data of Nutrient Elements in China’s National Standards for Surface Water Environmental Quality (μg/L); Table S3: Concentrations of Nutrient Salts (mg/L), OM (g/Kg), pH, Salt (ppt), Rare Earth Elements (REEs) (μg/L), and Heavy Metal Elements (μg/L) in Surface Sediments of the Yitong River System, China; Table S4: Pearson’s Correlation Coefficients of Nutrient Salts, OM, Ph, Salt, Rare Earth Elements (REEs), and Heavy Metals in Surface Sediments of the Yitong River System, China; Table S5: RQ values for each rare earth element at each sampling site in the intertidal surface sediments from the Yitong River system, China; Table S6: Data related to the exposure and acute toxicity of nutrients and REEs, including information on transformed-normal distribution, calculated means, standard deviations, and results of the Kolmogorov-Smirnov test (K-S test). References [17,19,32,33,34,35,36,37,38] are cited in the Supplementary Materials.
