# Supplementary material for: Bioavailability, Ecological Risk, and Microbial Response of Rare Earth Elements in Sediments of the Remediated Yitong River: An Integrated DGT and Multi-Parameter Assessment
Source: Microorganisms. 2025 Oct 24;13(11):2443. doi: 10.3390/microorganisms13112443 (PMC12654768; doi:10.3390/microorganisms13112443)
Supplement: Supplementary file 1 [file microorganisms-13-02443-s001.zip › microorganisms-3816609-supplementary.pdf]

Contens

Text S1. Experimental Methods 2

S1.1 DGT-Labile REE Calculation.. 2

S1.2 Species Sensitivity Distributions (SSD)... 2

S1.3 SPI Model for Mixture Risk.. 2

S1.3.1 Data Integration... 2

S1.3.2 Single-Element Risk Probability.. 2

S1.3.3 Joint Risk via Inclusion-Exclusion Principle 2

2. Supplementary tables 3

Table S1 REE-acute-toxicity data and the corresponding values of Predicted No Effect Concentration (PNEC)... 3

Table S2 Acute Toxicity Data of Nutrient Elements in China's National Standards for Surface Water Environmental Quality (μg/L) 4

Table S3 Concentrations of Nutrient Salts(mg/L), OM(g/Kg), pH, Salt(ppt), Rare Earth Elements (REEs)(μg/L), and Heavy Metal Elements(μg/L) in Surface Sediments of the Yitong River System, China 5

Table S4 Pearson's Correlation Coefficients of Nutrient Salts, OM, Ph, Salt, Rare Earth Elements (REEs), and Heavy Metals in Surface Sediments of the Yitong River System, China 6

Table S5 RQ values for each rare earth element at each sampling site in the intertidal surface sediments from the Yitong River system, China.. 7

Table S6 Data related to the exposure and acute toxicity of nutrients and REEs, including information on transformed-normal distribution, calculated means, standard deviations, and results of the Kolmogorov-Smirnov test (K-S test)....................................... 8

References..9

Text S1. Experimental Methods

S1.1 DGT-Labile REE Calculation

The mass of REEs (M) captured by DGT was calculated as:

$$M = \frac{C_e\left(V_e + V_g\right)}{f_e} \qquad (S1)$$

where C_e= eluent concentration, V_e = eluent volume (mL), Vg = gel volume (0.2 mL), and f_e = element-specific extraction efficiency (Table S1). C_{DGT} was then derived via:

$$C_{DGT} = \frac{M \triangle g}{DAt} \qquad (S2)$$

Where Δg = diffusion layer thickness (0.8 mm), D = temperature-dependent diffusion coefficient (EasySensor Ltd.), A = exposure area (3.14 cm^2), and t = deployment time (48 h).

S1.2 Species Sensitivity Distributions (SSD)

PNEC values (Table S1) were established per USEPA guidelines (USEPA, 2001, 2018): Endpoints (lethality/growth/reproduction) were selected. Acute toxicity data for 4 trophic levels (Table S1) were compiled. Toxicity distributions were fitted to log-normal probability densities.

S1.3 SPI Model for Mixture Risk

The SPI model quantified joint risks in three steps (Gu et al., 2025):

S1.3.1 Data Integration

DGT concentrations and toxicity thresholds were log-transformed; normality was confirmed via Kolmogorov-Smirnov tests (p>0.05).

S1.3.2 Single-Element Risk Probability

For each REE, the risk probability ($\Phi[Ai]$) was calculated as the overlap between exposure and SSD probability densities using PRA (Gu et al., 2025).

S1.3.3 Joint Risk via Inclusion-Exclusion Principle

The combined risk probability for n REEs was:

$$\Phi[A_1 + A_2 + \cdots + A_n] = \sum_{i-1}^{n} \Phi[A_i] - \sum_{i1<i2} \Phi[A_{i1}A_{i2}] + \cdots + (-1)^{r+1} \sum_{i1<i2\ldots<ir} \Phi[A_{i1}A_{i2} \ldots A_{ir}] + \cdots + (-1)^{n+1}\Phi[A_1 + A_2 + \cdots + A_n] \qquad (S3)$$

where r = subset size, avoiding overestimation of cumulative risks.

2. Supplementary tables

Table S1 REE-acute-toxicity data and the corresponding values of Predicted No Effect Concentration (PNEC)

Element	Algae		Crustaceans		Fish		Benthos	PNEC[a]
	Skeletonema costatum	Chlorella autotrophica	Daphnia magna	Hyalella azteca	Oncorhynchus mykiss	Danio rerio	Tubifex	
	EC50	EC50	LC50	LC50	LC50	LC50	EC50	
Y	3885.01	n.a.	n.a.	549.00	80640.28	n.a.	7201.79	0.549
La	4054.49	4051.71	24029.70	1665.00	68311.02	83812.26	10556.40	1.665
Ce	4158.17	4030.68	21960.68	651.00	54330.78	n.a.	11936.52	0.651
Pr	n.a.	n.a.	9003.51	441.00	n.a.	n.a.	10637.95	0.441
Nd	4375.03	4158.73	1384.32	511.00	34752.20	n.a.	10382.40	0.511
Sm	4313.47	4348.06	7580.16	846.00	67243.84	n.a.	10362.56	0.846
Eu	4432.32	n.a.	n.a.	717.00	n.a.	n.a.	11156.80	0.717
Gd	4685.97	4556.98	6716.71	599.00	36619.44	64901.98	11577.28	0.599
Tb	4533.42	4754.29	n.a.	693.00	n.a.	n.a.	13442.94	0.693
Dy	4593.88	4719.00	9165.00	897.00	n.a.	n.a.	13585.00	0.897
Ho	4829.92	4945.35	n.a.	755.00	n.a.	n.a.	11773.86	0.755
Er	4794.82	4759.69	n.a.	929.00	81809.70	n.a.	13835.71	0.929
Tm	4866.01	4938.64	n.a.	739.00	n.a.	n.a.	13934.25	0.739
Yb	4935.69	5011.81	n.a.	278.00	n.a.	46415.90	13857.30	0.278
Lu	5008.50	5152.00	n.a.	1054.00	n.a.	n.a.	15400.00	1.054
References	Tai et al. (2010)	Su et al. (2005)	Gonzalez et al. (2014)	Borgmann et al. (2005)	Dub´e et al. (2019)	Cui (2011)	Rucki et al. (2020)	

Table S2 Acute Toxicity Data of Nutrient Elements in China's National Standards for Surface Water Environmental Quality (μg/L)

Organism	Duration	End point	PO_4-P	NO_3-N	NH_4-N
Fish	96h	LC50	2.47E+07	2.07E+06	7.81E+09
Daphnid	48h	LC50	1.09E+07	9.95E+05	2.46E+09
Green algae	96h	EC50	2.81E+06	3.74E+05	1.61E+08

Table S3 Concentrations of Nutrient Salts(mg/L), OM(g/Kg), pH, Salt(ppt), Rare Earth Elements (REEs)(μg/L), and Heavy Metal Elements(μg/L) in Surface Sediments of the Yitong River System, China

Site		NH_4^+	NO_3^-	PO_4^{3-}	OM	Ph	Salt	Y	La	Ce	Pr	Nd	Sm
1	Mean	0.3159	0.3256	0.0960	45.3253	7.5900	0.0500	0.3599	0.1805	0.3300	0.0607	0.1752	0.0668
	SD	0.0020	0.0028	0.0019	0.9261	0.0557	0.0000	0.0056	0.0034	0.0116	0.0059	0.0044	0.0014
	Mean ± SD	0.3159±0.0020	0.3256±0.0028	0.0960±0.0019	45.3253±0.9261	7.5900±0.0557	0.0500±0.0000	0.3599±0.0056	0.1805±0.0034	0.3300±0.0116	0.0607±0.0059	0.1752±0.0044	0.0668±0.0014
2	Mean	0.3165	0.4668	0.0390	49.9387	7.3167	0.0400	0.2108	0.0929	0.1489	0.0281	0.0806	0.0276
	SD	0.0046	0.0044	0.0009	0.7144	0.1877	0.0000	0.0058	0.0029	0.0064	0.0021	0.0032	0.0004
	Mean ± SD	0.3165±0.0046	0.4668±0.0044	0.0390±0.0009	49.9387±0.7144	7.3167±0.1877	0.0400±0.0000	0.2108±0.0058	0.0929±0.0029	0.1489±0.0064	0.0281±0.0021	0.0806±0.0032	0.0276±0.0004
3	Mean	0.1389	0.3297	0.1106	30.3790	8.3967	0.0367	0.2729	0.2114	0.3949	0.0570	0.1786	0.0419
	SD	0.0036	0.0029	0.0014	0.4134	0.0416	0.0058	0.0037	0.0127	0.0174	0.0040	0.0035	0.0010
	Mean ± SD	0.1389±0.0036	0.3297±0.0029	0.1106±0.0014	30.3790±0.4134	8.3967±0.0416	0.0367±0.0058	0.2729±0.0037	0.2114±0.0127	0.3949±0.0174	0.0570±0.0040	0.1786±0.0035	0.0419±0.0010
4	Mean	0.2309	0.3539	0.0910	32.4097	7.9800	0.0333	0.2509	0.1244	0.2191	0.0331	0.1167	0.0348
	SD	0.0004	0.0024	0.0017	2.0410	0.0819	0.0058	0.0029	0.0055	0.0074	0.0034	0.0049	0.0006
	Mean ± SD	0.2309±0.0004	0.3539±0.0024	0.0910±0.0017	32.4097±2.0410	7.9800±0.0819	0.0333±0.0058	0.2509±0.0029	0.1244±0.0055	0.2191±0.0074	0.0331±0.0034	0.1167±0.0049	0.0348±0.0006
5	Mean	0.0466	0.2607	0.0194	60.7853	7.4100	0.0367	0.1856	0.0686	0.1229	0.0178	0.0590	0.0178
	SD	0.0010	0.0013	0.0003	1.2056	0.2512	0.0115	0.0085	0.0042	0.0040	0.0011	0.0017	0.0001
	Mean ± SD	0.0466±0.0010	0.2607±0.0013	0.0194±0.0003	60.7853±1.2056	7.4100±0.2512	0.0367±0.0115	0.1856±0.0085	0.0686±0.0042	0.1229±0.0040	0.0178±0.0011	0.0590±0.0017	0.0178±0.0001
6	Mean	0.4458	0.3389	0.0348	21.5660	8.8900	0.0367	0.2426	0.0950	0.1753	0.0171	0.0859	0.0166
	SD	0.0085	0.0052	0.0005	1.1191	0.0721	0.0058	0.0040	0.0040	0.0058	0.0012	0.0032	0.0005
	Mean ± SD	0.4458±0.0085	0.3389±0.0052	0.0348±0.0005	21.5660±1.1191	8.8900±0.0721	0.0367±0.0058	0.2426±0.0040	0.0950±0.0040	0.1753±0.0058	0.0171±0.0012	0.0859±0.0032	0.0166±0.0005
7	Mean	0.0251	0.2645	0.0103	12.9290	9.2633	0.0367	0.1857	0.0843	0.1450	0.0184	0.0582	0.0151
	SD	0.0008	0.0027	0.0002	0.6286	0.0611	0.0058	0.0175	0.0073	0.0136	0.0040	0.0125	0.0012
	Mean ± SD	0.0251±0.0008	0.2645±0.0027	0.0103±0.0002	12.9290±0.6286	9.2633±0.0611	0.0367±0.0058	0.1857±0.0175	0.0843±0.0073	0.1450±0.0136	0.0184±0.0040	0.0582±0.0125	0.0151±0.0012
8	Mean	0.1716	0.2978	0.0656	33.0437	9.3667	0.0400	0.1549	0.0516	0.0935	0.0121	0.0324	0.0041
	SD	0.0028	0.0020	0.0010	0.2224	0.0153	0.0173	0.0036	0.0018	0.0027	0.0005	0.0012	0.0001
	Mean ± SD	0.1716±0.0028	0.2978±0.0020	0.0656±0.0010	33.0437±0.2224	9.3667±0.0153	0.0400±0.0173	0.1549±0.0036	0.0516±0.0018	0.0935±0.0027	0.0121±0.0005	0.0324±0.0012	0.0041±0.0001
9	Mean	0.1315	0.2343	0.0098	32.9490	8.6700	0.0767	0.1637	0.0671	0.1207	0.0127	0.0422	0.0137
	SD	0.0011	0.0022	0.0002	0.4431	0.0265	0.0058	0.0045	0.0023	0.0076	0.0013	0.0012	0.0004
	Mean ± SD	0.1315±0.0011	0.2343±0.0022	0.0098±0.0002	32.9490±0.4431	8.6700±0.0265	0.0767±0.0058	0.1637±0.0045	0.0671±0.0023	0.1207±0.0076	0.0127±0.0013	0.0422±0.0012	0.0137±0.0004
10	Mean	0.1904	0.2973	0.0902	23.7490	9.1467	0.0800	0.1497	0.0438	0.0812	0.0080	0.0333	0.0084
	SD	0.0104	0.0038	0.0021	2.7719	0.1079	0.0000	0.0036	0.0046	0.0064	0.0009	0.0031	0.0004
	Mean ± SD	0.1904±0.0104	0.2973±0.0038	0.0902±0.0021	23.7490±2.7719	9.1467±0.1079	0.0800±0.0000	0.1497±0.0036	0.0438±0.0046	0.0812±0.0064	0.0080±0.0009	0.0333±0.0031	0.0084±0.0004
11	Mean	0.1907	0.4092	0.0881	29.8037	7.6133	0.1700	0.1628	0.0597	0.1177	0.0135	0.0551	0.0142
	SD	0.0006	0.0044	0.0011	0.6302	0.0723	0.0173	0.0043	0.0029	0.0040	0.0009	0.0031	0.0001
	Mean ± SD	0.1907±0.0006	0.4092±0.0044	0.0881±0.0011	29.8037±0.6302	7.6133±0.0723	0.1700±0.0173	0.1628±0.0043	0.0597±0.0029	0.1177±0.0040	0.0135±0.0009	0.0551±0.0031	0.0142±0.0001
12	Mean	0.2887	0.2877	0.2572	45.6020	8.1433	0.0600	0.1941	0.0976	0.1751	0.0191	0.0852	0.0098
	SD	0.0051	0.0017	0.0019	1.2539	0.0208	0.0000	0.0070	0.0026	0.0130	0.0012	0.0022	0.0002
	Mean ± SD	0.2887±0.0051	0.2877±0.0017	0.2572±0.0019	45.6020±1.2539	8.1433±0.0208	0.0600±0.0000	0.1941±0.0070	0.0976±0.0026	0.1751±0.0130	0.0191±0.0012	0.0852±0.0022	0.0098±0.0002
13	Mean	0.3433	0.2675	0.1506	60.8740	7.5400	0.0367	0.1789	0.0518	0.0900	0.0107	0.0327	0.0102
	SD	0.0036	0.0043	0.0127	0.4075	0.1114	0.0058	0.0172	0.0069	0.0114	0.0020	0.0000	0.0000
	Mean ± SD	0.3433±0.0036	0.2675±0.0043	0.1506±0.0127	60.8740±0.4075	7.5400±0.1114	0.0367±0.0058	0.1789±0.0172	0.0518±0.0069	0.0900±0.0114	0.0107±0.0020	0.0327±0.0000	0.0102±0.0000
14	Mean	0.6395	0.3080	0.0552	77.6297	8.4700	0.0433	0.2036	0.0786	0.1590	0.0167	0.0579	0.0124
	SD	0.0077	0.0019	0.0013	0.0753	0.2685	0.0058	0.0048	0.0018	0.0055	0.0011	0.0014	0.0003
	Mean ± SD	0.6395±0.0077	0.3080±0.0019	0.0552±0.0013	77.6297±0.0753	8.4700±0.2685	0.0433±0.0058	0.2036±0.0048	0.0786±0.0018	0.1590±0.0055	0.0167±0.0011	0.0579±0.0014	0.0124±0.0003
15	Mean	0.2061	0.2846	0.1038	31.5137	8.4467	0.1233	0.1507	0.0455	0.0967	0.0087	0.0400	0.0075
	SD	0.0040	0.0007	0.0016	1.1994	0.1102	0.0058	0.0028	0.0013	0.0027	0.0004	0.0011	0.0000
	Mean ± SD	0.2061±0.0040	0.2846±0.0007	0.1038±0.0016	31.5137±1.1994	8.4467±0.1102	0.1233±0.0058	0.1507±0.0028	0.0455±0.0013	0.0967±0.0027	0.0087±0.0004	0.0400±0.0011	0.0075±0.0000
16	Mean	0.2832	0.3096	0.1415	12.8870	9.2967	0.0767	0.2310	0.0837	0.1514	0.0168	0.0691	0.0133
	SD	0.0084	0.0011	0.0024	1.2333	0.0451	0.0115	0.0096	0.0066	0.0050	0.0009	0.0024	0.0003
	Mean ± SD	0.2832±0.0084	0.3096±0.0011	0.1415±0.0024	12.8870±1.2333	9.2967±0.0451	0.0767±0.0115	0.2310±0.0096	0.0837±0.0066	0.1514±0.0050	0.0168±0.0009	0.0691±0.0024	0.0133±0.0003
17	Mean	0.1796	0.3050	0.0288	16.2287	6.6433	0.0433	0.1675	0.0468	0.0891	0.0073	0.0386	0.0091
	SD	0.0028	0.0027	0.0001	0.5052	0.0907	0.0115	0.0042	0.0029	0.0014	0.0003	0.0013	0.0002
	Mean ± SD	0.1796±0.0028	0.3050±0.0027	0.0288±0.0001	16.2287±0.5052	6.6433±0.0907	0.0433±0.0115	0.1675±0.0042	0.0468±0.0029	0.0891±0.0014	0.0073±0.0003	0.0386±0.0013	0.0091±0.0002
18	Mean	0.4067	0.2888	0.2019	15.8177	6.7200	0.0700	0.1752	0.0431	0.0926	0.0090	0.0372	0.0083
	SD	0.0083	0.0026	0.0026	1.6505	0.0557	0.0000	0.0021	0.0019	0.0009	0.0011	0.0021	0.0001
	Mean ± SD	0.4067±0.0083	0.2888±0.0026	0.2019±0.0026	15.8177±1.6505	6.7200±0.0557	0.0700±0.0000	0.1752±0.0021	0.0431±0.0019	0.0926±0.0009	0.0090±0.0011	0.0372±0.0021	0.0083±0.0001
19	Mean	0.3572	0.2814	0.2194	23.3630	6.5133	0.1533	0.1928	0.0694	0.1276	0.0129	0.0546	0.0135
	SD	0.0038	0.0017	0.0064	0.3567	0.0351	0.0058	0.0059	0.0034	0.0048	0.0004	0.0008	0.0002
	Mean ± SD	0.3572±0.0038	0.2814±0.0017	0.2194±0.0064	23.3630±0.3567	6.5133±0.0351	0.1533±0.0058	0.1928±0.0059	0.0694±0.0034	0.1276±0.0048	0.0129±0.0004	0.0546±0.0008	0.0135±0.0002
20	Mean	0.1533	0.2744	0.0093	10.5230	8.0200	0.0467	0.1837	0.0597	0.1089	0.0101	0.0457	0.0069
	SD	0.0013	0.0025	0.0001	0.5008	0.0436	0.0058	0.0053	0.0033	0.0066	0.0003	0.0019	0.0002
	Mean ± SD	0.1533±0.0013	0.2744±0.0025	0.0093±0.0001	10.5230±0.5008	8.0200±0.0436	0.0467±0.0058	0.1837±0.0053	0.0597±0.0033	0.1089±0.0066	0.0101±0.0003	0.0457±0.0019	0.0069±0.0002
21	Mean	0.4957	0.2696	0.1521	31.3193	6.6900	0.2000	0.1812	0.0708	0.1346	0.0133	0.0537	0.0127
	SD	0.0038	0.0032	0.0029	0.3900	0.0557	0.0000	0.0042	0.0002	0.0063	0.0005	0.0013	0.0002
	Mean ± SD	0.4957±0.0038	0.2696±0.0032	0.1521±0.0029	31.3193±0.3900	6.6900±0.0557	0.2000±0.0000	0.1812±0.0042	0.0708±0.0002	0.1346±0.0063	0.0133±0.0005	0.0537±0.0013	0.0127±0.0002
22	Mean	0.2934	0.5407	0.0739	30.5377	9.4867	0.1500	0.2669	0.1719	0.2974	0.0421	0.1564	0.0326
	SD	0.0142	0.0189	0.0016	1.0925	0.0777	0.0000	0.0035	0.0081	0.0075	0.0053	0.0052	0.0025
	Mean ± SD	0.2934±0.0142	0.5407±0.0189	0.0739±0.0016	30.5377±1.0925	9.4867±0.0777	0.1500±0.0000	0.2669±0.0035	0.1719±0.0081	0.2974±0.0075	0.0421±0.0053	0.1564±0.0052	0.0326±0.0025

Site		Eu	Gd	Tb	Dy	Ho	Er	Tm	Yb	Lu	Ni	Cu	Zn	Cd
1	Mean	0.0718	0.0701	0.0522	0.0648	0.0369	0.0658	0.0328	0.0662	0.0530	3.4513	0.9887	10.3648	0.9438
	SD	0.0031	0.0072	0.0056	0.0016	0.0004	0.0032	0.0013	0.0013	0.0018	0.0428	0.0234	0.2173	0.0152
	Mean ± SD	0.0718±0.0031	0.0701±0.0072	0.0522±0.0056	0.0648±0.0016	0.0369±0.0004	0.0658±0.0032	0.0328±0.0013	0.0662±0.0013	0.0530±0.0018	3.4513±0.0428	0.9887±0.0234	10.3648±0.2173	0.9438±0.0152
2	Mean	0.0278	0.0338	0.0264	0.0325	0.0195	0.0564	0.0190	0.0443	0.0319	0.6982	0.7548	8.8158	0.6572
	SD	0.0013	0.0029	0.0026	0.0011	0.0016	0.0013	0.0004	0.0026	0.0010	0.0106	0.0518	0.1594	0.0163
	Mean ± SD	0.0278±0.0013	0.0338±0.0029	0.0264±0.0026	0.0325±0.0011	0.0195±0.0016	0.0564±0.0013	0.0190±0.0004	0.0443±0.0026	0.0319±0.0010	0.6982±0.0106	0.7548±0.0518	8.8158±0.1594	0.6572±0.0163
3	Mean	0.0282	0.0486	0.0247	0.0518	0.0183	0.0472	0.0154	0.0486	0.0318	2.1211	0.7020	5.5687	0.5177
	SD	0.0012	0.0030	0.0025	0.0013	0.0006	0.0010	0.0007	0.0015	0.0011	0.0387	0.0622	0.0847	0.0064
	Mean ± SD	0.0282±0.0012	0.0486±0.0030	0.0247±0.0025	0.0518±0.0013	0.0183±0.0006	0.0472±0.0010	0.0154±0.0007	0.0486±0.0015	0.0318±0.0011	2.1211±0.0387	0.7020±0.0622	5.5687±0.0847	0.5177±0.0064
4	Mean	0.0260	0.0357	0.0213	0.0387	0.0162	0.0398	0.0142	0.0483	0.0236	1.1989	0.6898	5.4846	0.4947
	SD	0.0008	0.0022	0.0018	0.0025	0.0007	0.0004	0.0006	0.0026	0.0007	0.0098	0.0729	0.1123	0.0026
	Mean ± SD	0.0260±0.0008	0.0357±0.0022	0.0213±0.0018	0.0387±0.0025	0.0162±0.0007	0.0398±0.0004	0.0142±0.0006	0.0483±0.0026	0.0236±0.0007	1.1989±0.0098	0.6898±0.0729	5.4846±0.1123	0.4947±0.0026
5	Mean	0.0172	0.0226	0.0151	0.0243	0.0132	0.0405	0.0099	0.0326	0.0206	1.0062	0.7042	7.1165	0.3351
	SD	0.0010	0.0012	0.0012	0.0007	0.0005	0.0018	0.0004	0.0020	0.0010	0.0194	0.0239	0.2248	0.0126
	Mean ± SD	0.0172±0.0010	0.0226±0.0012	0.0151±0.0012	0.0243±0.0007	0.0132±0.0005	0.0405±0.0018	0.0099±0.0004	0.0326±0.0020	0.0206±0.0010	1.0062±0.0194	0.7042±0.0239	7.1165±0.2248	0.3351±0.0126
6	Mean	0.0337	0.0281	0.0168	0.0310	0.0117	0.0424	0.0112	0.0393	0.0188	3.6523	0.5467	6.5960	0.3847
	SD	0.0010	0.0020	0.0011	0.0010	0.0011	0.0015	0.0002	0.0014	0.0007	0.0804	0.0343	0.1617	0.0149
	Mean ± SD	0.0337±0.0010	0.0281±0.0020	0.0168±0.0011	0.0310±0.0010	0.0117±0.0011	0.0424±0.0015	0.0112±0.0002	0.0393±0.0014	0.0188±0.0007	3.6523±0.0804	0.5467±0.0343	6.5960±0.1617	0.3847±0.0149
7	Mean	0.0152	0.0165	0.0113	0.0237	0.0089	0.0348	0.0066	0.0350	0.0162	0.8624	0.6107	6.0287	0.3743
	SD	0.0022	0.0032	0.0012	0.0020	0.0009	0.0030	0.0004	0.0013	0.0008	0.0570	0.0027	0.6389	0.0408
	Mean ± SD	0.0152±0.0022	0.0165±0.0032	0.0113±0.0012	0.0237±0.0020	0.0089±0.0009	0.0348±0.0030	0.0066±0.0004	0.0350±0.0013	0.0162±0.0008	0.8624±0.0570	0.6107±0.0027	6.0287±0.6389	0.3743±0.0408
8	Mean	0.0085	0.0097	0.0085	0.0188	0.0067	0.0291	0.0054	0.0249	0.0122	0.7062	0.5197	4.8662	0.3736
	SD	0.0005	0.0009	0.0008	0.0007	0.0004	0.0004	0.0002	0.0007	0.0012	0.0137	0.0282	0.0728	0.0132
	Mean ± SD	0.0085±0.0005	0.0097±0.0009	0.0085±0.0008	0.0188±0.0007	0.0067±0.0004	0.0291±0.0004	0.0054±0.0002	0.0249±0.0007	0.0122±0.0012	0.7062±0.0137	0.5197±0.0282	4.8662±0.0728	0.3736±0.0132
9	Mean	0.0113	0.0112	0.0095	0.0174	0.0074	0.0268	0.0062	0.0257	0.0137	1.0810	0.5443	5.8616	0.3589
	SD	0.0005	0.0010	0.0009	0.0004	0.0003	0.0007	0.0001	0.0004	0.0006	0.0124	0.0345	0.1100	0.0125
	Mean ± SD	0.0113±0.0005	0.0112±0.0010	0.0095±0.0009	0.0174±0.0004	0.0074±0.0003	0.0268±0.0007	0.0062±0.0001	0.0257±0.0004	0.0137±0.0006	1.0810±0.0124	0.5443±0.0345	5.8616±0.1100	0.3589±0.0125
10	Mean	0.0131	0.0111	0.0066	0.0171	0.0056	0.0280	0.0060	0.0284	0.0125	1.1695	0.5244	6.2990	0.2966
	SD	0.0004	0.0017	0.0015	0.0000	0.0000	0.0011	0.0004	0.0044	0.0011	0.0336	0.0224	0.3811	0.0353
	Mean ± SD	0.0131±0.0004	0.0111±0.0017	0.0066±0.0015	0.0171±0.0000	0.0056±0.0000	0.0280±0.0011	0.0060±0.0004	0.0284±0.0044	0.0125±0.0011	1.1695±0.0336	0.5244±0.0224	6.2990±0.3811	0.2966±0.0353
11	Mean	0.0155	0.0126	0.0069	0.0202	0.0065	0.0324	0.0064	0.0256	0.0137	0.6345	0.4188	5.2322	0.3168
	SD	0.0012	0.0015	0.0002	0.0011	0.0002	0.0010	0.0001	0.0008	0.0011	0.0201	0.0347	0.1350	0.0133
	Mean ± SD	0.0155±0.0012	0.0126±0.0015	0.0069±0.0002	0.0202±0.0011	0.0065±0.0002	0.0324±0.0010	0.0064±0.0001	0.0256±0.0008	0.0137±0.0011	0.6345±0.0201	0.4188±0.0347	5.2322±0.1350	0.3168±0.0133
12	Mean	0.0165	0.0255	0.0072	0.0247	0.0070	0.0289	0.0057	0.0290	0.0120	1.9380	0.4652	6.2025	0.3004
	SD	0.0005	0.0021	0.0006	0.0015	0.0005	0.0009	0.0001	0.0004	0.0008	0.0231	0.0061	0.1751	0.0101
	Mean ± SD	0.0165±0.0005	0.0255±0.0021	0.0072±0.0006	0.0247±0.0015	0.0070±0.0005	0.0289±0.0009	0.0057±0.0001	0.0290±0.0004	0.0120±0.0008	1.9380±0.0231	0.4652±0.0061	6.2025±0.1751	0.3004±0.0101
13	Mean	0.0190	0.0139	0.0059	0.0186	0.0070	0.0364	0.0059	0.0307	0.0131	1.7283	0.6234	5.6162	0.2734
	SD	0.0007	0.0023	0.0023	0.0018	0.0014	0.0050	0.0004	0.0000	0.0015	0.0656	0.3282	0.2572	0.0013
	Mean ± SD	0.0190±0.0007	0.0139±0.0023	0.0059±0.0023	0.0186±0.0018	0.0070±0.0014	0.0364±0.0050	0.0059±0.0004	0.0307±0.0000	0.0131±0.0015	1.7283±0.0656	0.6234±0.3282	5.6162±0.2572	0.2734±0.0013
14	Mean	0.0144	0.0189	0.0070	0.0232	0.0073	0.0395	0.0047	0.0390	0.0115	0.5918	0.5036	6.5656	0.2809
	SD	0.0004	0.0014	0.0010	0.0004	0.0002	0.0005	0.0002	0.0015	0.0003	0.0077	0.0373	0.0793	0.0135
	Mean ± SD	0.0144±0.0004	0.0189±0.0014	0.0070±0.0010	0.0232±0.0004	0.0073±0.0002	0.0395±0.0005	0.0047±0.0002	0.0390±0.0015	0.0115±0.0003	0.5918±0.0077	0.5036±0.0373	6.5656±0.0793	0.2809±0.0135
15	Mean	0.0146	0.0136	0.0061	0.0203	0.0063	0.0299	0.0042	0.0285	0.0096	0.7363	0.4257	5.3944	0.2669
	SD	0.0002	0.0003	0.0004	0.0008	0.0002	0.0011	0.0001	0.0012	0.0005	0.0275	0.0183	0.1426	0.0166
	Mean ± SD	0.0146±0.0002	0.0136±0.0003	0.0061±0.0004	0.0203±0.0008	0.0063±0.0002	0.0299±0.0011	0.0042±0.0001	0.0285±0.0012	0.0096±0.0005	0.7363±0.0275	0.4257±0.0183	5.3944±0.1426	0.2669±0.0166
16	Mean	0.0241	0.0214	0.0053	0.0261	0.0071	0.0388	0.0064	0.0340	0.0112	1.1918	0.4479	4.3742	0.2375
	SD	0.0012	0.0023	0.0005	0.0017	0.0003	0.0017	0.0003	0.0010	0.0005	0.0295	0.0131	0.2279	0.0093
	Mean ± SD	0.0241±0.0012	0.0214±0.0023	0.0053±0.0005	0.0261±0.0017	0.0071±0.0003	0.0388±0.0017	0.0064±0.0003	0.0340±0.0010	0.0112±0.0005	1.1918±0.0295	0.4479±0.0131	4.3742±0.2279	0.2375±0.0093
17	Mean	0.0155	0.0140	0.0050	0.0190	0.0046	0.0277	0.0041	0.0269	0.0090	0.4077	0.4181	4.2959	0.2214
	SD	0.0009	0.0007	0.0006	0.0003	0.0003	0.0010	0.0001	0.0011	0.0002	0.0088	0.0341	0.1123	0.0114
	Mean ± SD	0.0155±0.0009	0.0140±0.0007	0.0050±0.0006	0.0190±0.0003	0.0046±0.0003	0.0277±0.0010	0.0041±0.0001	0.0269±0.0011	0.0090±0.0002	0.4077±0.0088	0.4181±0.0341	4.2959±0.1123	0.2214±0.0114
18	Mean	0.0097	0.0123	0.0044	0.0202	0.0048	0.0321	0.0042	0.0320	0.0090	1.4126	0.4018	5.6763	0.1989
	SD	0.0005	0.0006	0.0005	0.0005	0.0004	0.0013	0.0003	0.0008	0.0001	0.0125	0.0191	0.0446	0.0019
	Mean ± SD	0.0097±0.0005	0.0123±0.0006	0.0044±0.0005	0.0202±0.0005	0.0048±0.0004	0.0321±0.0013	0.0042±0.0003	0.0320±0.0008	0.0090±0.0001	1.4126±0.0125	0.4018±0.0191	5.6763±0.0446	0.1989±0.0019
19	Mean	0.0134	0.0190	0.0052	0.0201	0.0063	0.0369	0.0056	0.0339	0.0094	1.1589	0.4878	5.7347	0.2286
	SD	0.0003	0.0014	0.0004	0.0013	0.0006	0.0014	0.0004	0.0013	0.0004	0.0082	0.0381	0.2019	0.0069
	Mean ± SD	0.0134±0.0003	0.0190±0.0014	0.0052±0.0004	0.0201±0.0013	0.0063±0.0006	0.0369±0.0014	0.0056±0.0004	0.0339±0.0013	0.0094±0.0004	1.1589±0.0082	0.4878±0.0381	5.7347±0.2019	0.2286±0.0069
20	Mean	0.0074	0.0149	0.0043	0.0204	0.0062	0.0404	0.0057	0.0357	0.0143	0.6596	0.4346	6.1961	0.1948
	SD	0.0003	0.0008	0.0006	0.0006	0.0003	0.0008	0.0001	0.0024	0.0009	0.0065	0.0269	0.2968	0.0047
	Mean ± SD	0.0074±0.0003	0.0149±0.0008	0.0043±0.0006	0.0204±0.0006	0.0062±0.0003	0.0404±0.0008	0.0057±0.0001	0.0357±0.0024	0.0143±0.0009	0.6596±0.0065	0.4346±0.0269	6.1961±0.2968	0.1948±0.0047
21	Mean	0.0124	0.0133	0.0040	0.0243	0.0067	0.0316	0.0049	0.0314	0.0095	0.6410	0.3670	5.4496	0.2375
	SD	0.0005	0.0015	0.0004	0.0015	0.0003	0.0004	0.0003	0.0008	0.0004	0.0158	0.0259	0.1178	0.0042
	Mean ± SD	0.0124±0.0005	0.0133±0.0015	0.0040±0.0004	0.0243±0.0015	0.0067±0.0003	0.0316±0.0004	0.0049±0.0003	0.0314±0.0008	0.0095±0.0004	0.6410±0.0158	0.3670±0.0259	5.4496±0.1178	0.2375±0.0042
22	Mean	0.0241	0.0415	0.0091	0.0346	0.0105	0.0492	0.0053	0.0352	0.0109	0.9196	0.4467	5.6819	0.2108
	SD	0.0032	0.0030	0.0007	0.0040	0.0021	0.0016	0.0008	0.0042	0.0017	0.0320	0.0056	0.0279	0.0123
	Mean ± SD	0.0241±0.0032	0.0415±0.0030	0.0091±0.0007	0.0346±0.0040	0.0105±0.0021	0.0492±0.0016	0.0053±0.0008	0.0352±0.0042	0.0109±0.0017	0.9196±0.0320	0.4467±0.0056	5.6819±0.0279	0.2108±0.0123

Site		Pb	Co	Mn	Cr	As	Sb	V	Mo	W	Se	Fe
1	Mean	0.8792	4.1948	5567.7111	0.3477	24.0496	0.8328	0.2127	2.0824	0.3211	0.1618	8004.1334
	SD	0.0053	0.0457	17.7033	0.0080	0.0820	0.0205	0.0180	0.0545	0.0064	0.0012	51.2335
	Mean ± SD	0.8792±0.0053	4.1948±0.0457	5567.7111±17.7033	0.3477±0.0080	24.0496±0.0820	0.8328±0.0205	0.2127±0.0180	2.0824±0.0545	0.3211±0.0064	0.1618±0.0012	8004.1334±51.2335
2	Mean	0.5860	0.6148	1490.6381	0.3064	10.7911	0.7727	0.1903	0.9633	0.1681	0.1404	5940.1736
	SD	0.0064	0.0082	4.8704	0.0110	0.1107	0.0046	0.0166	0.0334	0.0071	0.0051	34.1446
	Mean ± SD	0.5860±0.0064	0.6148±0.0082	1490.6381±4.8704	0.3064±0.0110	10.7911±0.1107	0.7727±0.0046	0.1903±0.0166	0.9633±0.0334	0.1681±0.0071	0.1404±0.0051	5940.1736±34.1446
3	Mean	0.5551	2.4005	1024.4745	0.3470	1.5210	0.7943	0.0985	1.1750	0.1094	0.1607	5919.4325
	SD	0.0027	0.0406	2.7649	0.0180	0.0733	0.0125	0.0135	0.0192	0.0050	0.0029	26.4407
	Mean ± SD	0.5551±0.0027	2.4005±0.0406	1024.4745±2.7649	0.3470±0.0180	1.5210±0.0733	0.7943±0.0125	0.0985±0.0135	1.1750±0.0192	0.1094±0.0050	0.1607±0.0029	5919.4325±26.4407
4	Mean	0.4635	1.5537	1720.0865	0.3123	10.2096	0.8955	0.4153	2.1783	0.1841	0.1809	3826.0198
	SD	0.0085	0.0147	5.9879	0.0122	0.1533	0.0233	0.0267	0.0812	0.0083	0.0013	23.3707
	Mean ± SD	0.4635±0.0085	1.5537±0.0147	1720.0865±5.9879	0.3123±0.0122	10.2096±0.1533	0.8955±0.0233	0.4153±0.0267	2.1783±0.0812	0.1841±0.0083	0.1809±0.0013	3826.0198±23.3707
5	Mean	0.3835	0.3493	99.8376	0.3092	0.6089	0.8850	0.3296	1.2069	0.1058	0.1571	9384.9003
	SD	0.0010	0.0015	0.3149	0.0028	0.0179	0.0133	0.0119	0.0445	0.0013	0.0023	38.3695
	Mean ± SD	0.3835±0.0010	0.3493±0.0015	99.8376±0.3149	0.3092±0.0028	0.6089±0.0179	0.8850±0.0133	0.3296±0.0119	1.2069±0.0445	0.1058±0.0013	0.1571±0.0023	9384.9003±38.3695
6	Mean	0.3907	4.9155	4143.2892	0.3300	16.2620	0.9659	0.2786	3.8403	0.2256	0.1992	5809.1321
	SD	0.0043	0.0327	18.9487	0.0065	0.3067	0.0268	0.0056	0.0144	0.0096	0.0053	17.3578
	Mean ± SD	0.3907±0.0043	4.9155±0.0327	4143.2892±18.9487	0.3300±0.0065	16.2620±0.3067	0.9659±0.0268	0.2786±0.0056	3.8403±0.0144	0.2256±0.0096	0.1992±0.0053	5809.1321±17.3578
7	Mean	0.3476	0.2763	39.7477	0.2407	0.5294	0.9179	0.5845	1.5606	0.0871	0.1855	4873.6640
	SD	0.0096	0.0126	0.0285	0.0327	0.0118	0.0935	0.1409	0.1939	0.0238	0.0134	51.2415
	Mean ± SD	0.3476±0.0096	0.2763±0.0126	39.7477±0.0285	0.2407±0.0327	0.5294±0.0118	0.9179±0.0935	0.5845±0.1409	1.5606±0.1939	0.0871±0.0238	0.1855±0.0134	4873.6640±51.2415
8	Mean	0.3222	0.5497	1104.3609	0.2858	4.1939	0.7764	0.2899	1.1778	0.1368	0.2285	6024.2625
	SD	0.0007	0.0116	4.3947	0.0040	0.0999	0.0056	0.0082	0.0457	0.0057	0.0021	48.1522
	Mean ± SD	0.3222±0.0007	0.5497±0.0116	1104.3609±4.3947	0.2858±0.0040	4.1939±0.0999	0.7764±0.0056	0.2899±0.0082	1.1778±0.0457	0.1368±0.0057	0.2285±0.0021	6024.2625±48.1522
9	Mean	0.4186	1.1723	1630.1923	0.2365	4.0684	0.9400	0.1771	6.0526	0.1074	0.2436	6087.5220
	SD	0.0035	0.0081	2.1931	0.0123	0.2180	0.0081	0.0153	0.1508	0.0032	0.0045	34.7532
	Mean ± SD	0.4186±0.0035	1.1723±0.0081	1630.1923±2.1931	0.2365±0.0123	4.0684±0.2180	0.9400±0.0081	0.1771±0.0153	6.0526±0.1508	0.1074±0.0032	0.2436±0.0045	6087.5220±34.7532
10	Mean	0.3255	1.8728	1486.4755	0.2395	14.8271	0.7108	0.1538	1.2844	0.1338	0.2502	5061.3435
	SD	0.0040	0.0250	8.5163	0.0196	0.4007	0.0127	0.0167	0.1443	0.0006	0.0219	24.8774
	Mean ± SD	0.3255±0.0040	1.8728±0.0250	1486.4755±8.5163	0.2395±0.0196	14.8271±0.4007	0.7108±0.0127	0.1538±0.0167	1.2844±0.1443	0.1338±0.0006	0.2502±0.0219	5061.3435±24.8774
11	Mean	0.3081	0.8729	1101.8020	0.2750	25.9041	0.6789	0.1163	0.8669	0.1173	0.2511	12378.5178
	SD	0.0005	0.0038	6.0697	0.0094	0.4023	0.0135	0.0085	0.0118	0.0011	0.0063	51.4872
	Mean ± SD	0.3081±0.0005	0.8729±0.0038	1101.8020±6.0697	0.2750±0.0094	25.9041±0.4023	0.6789±0.0135	0.1163±0.0085	0.8669±0.0118	0.1173±0.0011	0.2511±0.0063	12378.5178±51.4872
12	Mean	0.3055	2.1972	2009.7258	0.2747	33.1107	0.7752	0.1973	2.4801	0.2625	0.3114	9707.3993
	SD	0.0041	0.0209	3.3636	0.0153	1.1181	0.0155	0.0102	0.0985	0.0078	0.0031	43.2205
	Mean ± SD	0.3055±0.0041	2.1972±0.0209	2009.7258±3.3636	0.2747±0.0153	33.1107±1.1181	0.7752±0.0155	0.1973±0.0102	2.4801±0.0985	0.2625±0.0078	0.3114±0.0031	9707.3993±43.2205
13	Mean	0.2811	1.5038	1110.8772	0.3230	18.7541	0.9717	0.2159	10.6731	0.2757	0.2971	6094.9440
	SD	0.0085	0.0320	14.3650	0.0333	0.6340	0.0081	0.0271	0.0138	0.0141	0.0023	108.9942
	Mean ± SD	0.2811±0.0085	1.5038±0.0320	1110.8772±14.3650	0.3230±0.0333	18.7541±0.6340	0.9717±0.0081	0.2159±0.0271	10.6731±0.0138	0.2757±0.0141	0.2971±0.0023	6094.9440±108.9942
14	Mean	0.2907	0.3107	751.2073	0.2526	4.3485	0.8244	0.2990	2.2219	0.0994	0.2976	2349.8839
	SD	0.0021	0.0039	0.6826	0.0132	0.1993	0.0047	0.0118	0.0858	0.0058	0.0060	8.1931
	Mean ± SD	0.2907±0.0021	0.3107±0.0039	751.2073±0.6826	0.2526±0.0132	4.3485±0.1993	0.8244±0.0047	0.2990±0.0118	2.2219±0.0858	0.0994±0.0058	0.2976±0.0060	2349.8839±8.1931
15	Mean	0.2675	0.4649	551.8001	0.8231	8.2645	0.8509	0.1409	2.8085	0.2398	0.2518	4048.6122
	SD	0.0026	0.0062	1.8862	0.0172	0.1033	0.0217	0.0086	0.1300	0.0197	0.0090	16.7205
	Mean ± SD	0.2675±0.0026	0.4649±0.0062	551.8001±1.8862	0.8231±0.0172	8.2645±0.1033	0.8509±0.0217	0.1409±0.0086	2.8085±0.1300	0.2398±0.0197	0.2518±0.0090	4048.6122±16.7205
16	Mean	0.4405	1.4870	1595.9269	0.2958	28.8866	0.8588	0.2267	2.4012	0.2217	0.2951	9026.9398
	SD	0.0017	0.0199	3.9992	0.0136	0.7267	0.0302	0.0136	0.0885	0.0025	0.0074	75.2496
	Mean ± SD	0.4405±0.0017	1.4870±0.0199	1595.9269±3.9992	0.2958±0.0136	28.8866±0.7267	0.8588±0.0302	0.2267±0.0136	2.4012±0.0885	0.2217±0.0025	0.2951±0.0074	9026.9398±75.2496
17	Mean	0.2391	0.1898	907.9777	0.3413	8.5448	0.8302	0.1511	1.9859	0.2222	0.3110	8186.8024
	SD	0.0018	0.0046	2.6934	0.0158	0.1556	0.0089	0.0015	0.0412	0.0096	0.0107	58.2396
	Mean ± SD	0.2391±0.0018	0.1898±0.0046	907.9777±2.6934	0.3413±0.0158	8.5448±0.1556	0.8302±0.0089	0.1511±0.0015	1.9859±0.0412	0.2222±0.0096	0.3110±0.0107	8186.8024±58.2396
18	Mean	0.2461	2.4051	812.1977	0.2463	10.1305	0.7452	0.1811	2.1235	0.4419	0.3058	6029.1117
	SD	0.0024	0.0011	3.9352	0.0121	0.3835	0.0256	0.0090	0.0918	0.0273	0.0052	17.2952
	Mean ± SD	0.2461±0.0024	2.4051±0.0011	812.1977±3.9352	0.2463±0.0121	10.1305±0.3835	0.7452±0.0256	0.1811±0.0090	2.1235±0.0918	0.4419±0.0273	0.3058±0.0052	6029.1117±17.2952
19	Mean	0.2593	1.8626	1170.5503	0.2888	16.0356	0.7953	0.2226	2.4395	0.3060	0.3367	6847.9280
	SD	0.0021	0.0165	4.6555	0.0147	0.4923	0.0143	0.0173	0.0877	0.0129	0.0015	24.9302
	Mean ± SD	0.2593±0.0021	1.8626±0.0165	1170.5503±4.6555	0.2888±0.0147	16.0356±0.4923	0.7953±0.0143	0.2226±0.0173	2.4395±0.0877	0.3060±0.0129	0.3367±0.0015	6847.9280±24.9302
20	Mean	0.2418	0.5888	1239.2551	0.2520	1.2698	0.8215	0.6264	2.0770	0.0581	0.2993	4899.4512
	SD	0.0010	0.0024	4.4052	0.0038	0.0371	0.0253	0.0531	0.1237	0.0038	0.0042	14.5265
	Mean ± SD	0.2418±0.0010	0.5888±0.0024	1239.2551±4.4052	0.2520±0.0038	1.2698±0.0371	0.8215±0.0253	0.6264±0.0531	2.0770±0.1237	0.0581±0.0038	0.2993±0.0042	4899.4512±14.5265
21	Mean	0.2145	0.5209	545.3637	0.2677	8.7160	0.8349	0.1580	1.9982	0.2482	0.2932	3834.2326
	SD	0.0017	0.0113	0.3957	0.0102	0.0788	0.0119	0.0126	0.1222	0.0120	0.0067	6.5469
	Mean ± SD	0.2145±0.0017	0.5209±0.0113	545.3637±0.3957	0.2677±0.0102	8.7160±0.0788	0.8349±0.0119	0.1580±0.0126	1.9982±0.1222	0.2482±0.0120	0.2932±0.0067	3834.2326±6.5469
22	Mean	0.2241	0.6459	372.1705	0.2903	1.2960	0.8422	0.4823	2.1325	0.0465	0.2917	5004.4328
	SD	0.0013	0.0158	3.5681	0.0238	0.2061	0.0704	0.0497	0.1839	0.0018	0.0172	73.8875
	Mean ± SD	0.2241±0.0013	0.6459±0.0158	372.1705±3.5681	0.2903±0.0238	1.2960±0.2061	0.8422±0.0704	0.4823±0.0497	2.1325±0.1839	0.0465±0.0018	0.2917±0.0172	5004.4328±73.8875

Table S4 Pearson's Correlation Coefficients of Nutrient Salts, OM, Ph, Salt, Rare Earth Elements (REEs), and Heavy Metals in Surface Sediments of the Yitong River System, China

	NH_4^+	NO_3^-	PO_4^{3-}	OM	pH	Salt	Y	La	Ce	Pr	Nd	Sm	Eu	Gd	Tb	Dy	Ho	Er	Tm	Yb	Lu	Ni	Cu	Zn	Cd	Pb	Co	Mn	Cr	As	Sb	V	Mo	W	Se	Fe
NH_4^+	1.000																																			
NO_3^-	0.133	1.000																																		
PO_4^{3-}	0.390	-0.114	1.000																																	
OM	0.346	0.049	0.008	1.000																																
Ph	-0.244	0.162	-0.331	-0.111	1.000																															
Salt	0.223	0.209	0.356	-0.197	-0.209	1.000																														
Y	0.184	0.397	0.020	0.115	0.087	-0.184	1.000																													
La	-0.004	.470*	0.011	0.105	0.210	-0.107	.885**	1.000																												
Ce	0.026	.429*	0.036	0.105	0.192	-0.091	.880**	.995**	1.000																											
Pr	-0.033	.456*	-0.020	0.178	0.126	-0.155	.906**	.969**	.963**	1.000																										
Nd	0.017	.517*	0.034	0.095	0.159	-0.082	.915**	.987**	.982**	.971**	1.000																									
Sm	-0.005	0.407	-0.070	0.183	-0.018	-0.135	.919**	.872**	.864**	.950**	.903**	1.000																								
Eu	0.129	0.298	-0.034	0.181	0.007	-0.212	.893**	.691**	.686**	.784**	.749**	.888**	1.000																							
Gd	0.060	.447*	0.008	0.194	0.041	-0.200	.958**	.912**	.902**	.958**	.946**	.961**	.894**	1.000																						
Tb	-0.050	0.252	-0.175	0.246	-0.030	-0.349	.813**	.696**	.684**	.825**	.734**	.915**	.912**	.883**	1.000																					
Dy	0.047	0.348	-0.008	0.137	0.030	-0.207	.943**	.903**	.906**	.959**	.929**	.963**	.888**	.973**	.901**	1.000																				
Ho	-0.018	0.285	-0.141	0.274	-0.052	-0.290	.849**	.729**	.716**	.855**	.769**	.939**	.921**	.913**	.991**	.922**	1.000																			
Er	0.161	.521*	-0.159	0.261	-0.025	-0.210	.857**	.726**	.701**	.805**	.758**	.844**	.816**	.874**	.822**	.831**	.867**	1.000																		
Tm	-0.015	0.250	-0.130	0.226	-0.097	-0.321	.801**	.653**	.640**	.790**	.698**	.896**	.912**	.865**	.988**	.883**	.985**	.838**	1.000																	
Yb	0.176	0.259	-0.061	0.191	-0.067	-0.319	.904**	.760**	.759**	.841**	.790**	.903**	.859**	.903**	.893**	.925**	.914**	.879**	.898**	1.000																
Lu	-0.099	0.215	-0.190	0.246	-0.057	-0.377	.778**	.679**	.668**	.812**	.708**	.891**	.873**	.857**	.984**	.885**	.978**	.828**	.986**	.888**	1.000															
Ni	0.196	-0.024	0.182	0.043	0.087	-0.298	.662**	.500*	.513*	.501*	.533*	.538**	.736**	.616**	.615**	.623**	.587**	.470*	.606**	.580**	.580**	1.000														
Cu	-0.176	0.097	-0.230	0.401	-0.003	-.524*	.658**	.563**	.536*	.703**	.572**	.790**	.770**	.742**	.913**	.745**	.901**	.736**	.900**	.793**	.921**	.519*	1.000													
Zn	0.136	0.180	-0.160	.429*	-0.134	-0.258	.550**	0.375	0.354	.516*	0.420	.638**	.703**	.638**	.796**	.590**	.801**	.755**	.810**	.705**	.800**	.478*	.769**	1.000												
Cd	-0.070	0.213	-0.182	0.277	-0.009	-0.359	.689**	.590**	.574**	.737**	.617**	.830**	.840**	.778**	.969**	.811**	.943**	.733**	.963**	.814**	.960**	.533*	.907**	.785**	1.000											
Pb	-0.106	0.149	-0.153	0.202	0.056	-0.394	.739**	.617**	.606**	.746**	.639**	.831**	.859**	.791**	.943**	.824**	.926**	.739**	.950**	.819**	.948**	.577**	.889**	.722**	.950**	1.000										
Co	0.273	-0.008	0.285	-0.103	0.019	-0.193	.581**	0.399	0.419	0.396	.445*	.452*	.647**	.518*	.519*	.533*	.482*	0.376	.531*	.514*	.480*	.954**	0.382	0.397	.448*	.503*	1.000									
Mn	0.234	0.051	0.037	0.021	0.028	-0.243	.642**	0.369	0.373	.432*	.434*	.560**	.805**	.597**	.696**	.599**	.663**	.509*	.721**	.617**	.655**	.836**	.530*	.596**	.671**	.693**	.842**	1.000								
Cr	-0.087	-0.024	0.033	0.029	0.019	0.125	-0.046	-0.036	-0.006	-0.001	0.011	0.015	0.108	0.050	0.058	0.065	0.058	-0.017	0.031	0.006	0.020	0.015	-0.020	-0.046	0.043	0.015	-0.045	-0.014	1.000							
As	0.259	0.027	.602**	0.016	-0.122	0.151	0.160	-0.033	-0.030	-0.013	0.038	0.059	0.332	0.121	0.099	0.089	0.099	0.020	0.178	0.052	0.092	0.402	0.010	0.094	0.142	0.216	.474*	.515*	-0.016	1.000						
Sb	-0.019	-0.268	-0.313	0.116	0.184	-0.330	0.179	0.078	0.052	0.019	0.036	0.068	0.164	0.066	0.084	0.059	0.089	0.080	0.044	0.122	0.045	0.291	0.226	0.000	0.028	0.070	0.121	0.129	0.120	-0.214	1.000					
V	-0.226	0.087	-0.420	-0.175	0.347	-0.244	0.117	0.089	0.044	0.036	0.067	-0.001	-0.096	0.034	-0.051	-0.015	-0.018	0.175	-0.080	0.118	-0.045	-0.149	0.031	0.041	-0.121	-0.154	-0.235	-0.161	-0.252	-.439*	0.338	1.000				
Mo	0.169	-0.294	0.121	0.257	-0.030	-0.133	-0.102	-0.185	-0.196	-0.208	-0.223	-0.159	-0.024	-0.179	-0.158	-0.207	-0.155	-0.146	-0.149	-0.158	-0.155	0.231	0.023	-0.104	-0.146	-0.120	0.152	0.101	0.054	0.151	.640**	-0.085	1.000			
W	.431*	-0.259	.695**	-0.074	-.565**	0.087	0.087	-0.163	-0.134	-0.094	-0.096	0.024	0.226	0.046	0.089	0.069	0.081	-0.022	0.135	0.122	0.041	0.384	-0.003	0.092	0.072	0.082	.486*	0.347	0.175	.518*	-0.052	-.472*	0.228	1.000		
Se	0.386	-0.189	.491*	-0.186	-0.190	.441*	-0.409	-.448*	-.424*	-.550**	-.451*	-.586**	-.515*	-.522*	-.744**	-.579**	-.705**	-.527*	-.708**	-.555**	-.754**	-0.324	-.786**	-.551**	-.770**	-.728**	-0.206	-0.287	-0.096	0.227	-0.159	-0.055	0.261	0.270	1.000	
Fe	-0.330	0.061	0.193	-0.065	-0.203	0.089	0.006	-0.036	-0.040	0.002	0.018	0.038	0.152	0.050	0.073	0.008	0.067	-0.021	0.111	-0.154	0.103	0.139	0.040	-0.001	0.075	0.166	0.143	0.181	-0.135	.596**	-0.295	-0.325	-0.108	0.136	-0.004	1.000

**. Correlation is significant at the 0.01 level (2-tailed).

*. Correlation is significant at the 0.05 level (2-tailed).

Table S5 RQ values for each rare earth element at each sampling site in the intertidal surface sediments from the Yitong River system, China.

Site	Y		La		Ce		Pr		Nd		Sm		Eu		Gd		Tb		Dy		Ho		Er		Tm		Yb		Lu	
	Mean	S.D.	Mean	S.D.	Mean	S.D.	Mean	S.D.	Mean	S.D.	Mean	S.D.	Mean	S.D.	Mean	S.D.	Mean	S.D.	Mean	S.D.	Mean	S.D.	Mean	S.D.	Mean	S.D.	Mean	S.D.	Mean	S.D.
YR1	0.6555	0.0101	0.1084	0.0020	0.5069	0.0178	0.1377	0.0133	0.3428	0.0085	0.0790	0.0016	0.1001	0.0044	0.1170	0.0120	0.0753	0.0081	0.0722	0.0018	0.0489	0.0006	0.0708	0.0035	0.0444	0.0018	0.2383	0.0045	0.0503	0.0017
YR2	0.3839	0.0106	0.0558	0.0017	0.2288	0.0098	0.0637	0.0048	0.1577	0.0063	0.0326	0.0005	0.0388	0.0018	0.0565	0.0048	0.0381	0.0037	0.0362	0.0012	0.0258	0.0022	0.0607	0.0014	0.0257	0.0006	0.1594	0.0094	0.0303	0.0010
YR3	0.4971	0.0068	0.1269	0.0076	0.6067	0.0268	0.1293	0.0091	0.3495	0.0069	0.0495	0.0011	0.0394	0.0017	0.0812	0.0050	0.0357	0.0037	0.0578	0.0014	0.0243	0.0008	0.0508	0.0010	0.0208	0.0009	0.1748	0.0052	0.0302	0.0010
YR4	0.4570	0.0053	0.0747	0.0033	0.3366	0.0113	0.0751	0.0077	0.2284	0.0095	0.0411	0.0008	0.0363	0.0011	0.0597	0.0037	0.0308	0.0026	0.0431	0.0028	0.0215	0.0009	0.0428	0.0004	0.0193	0.0008	0.1738	0.0093	0.0224	0.0007
YR5	0.3382	0.0155	0.0412	0.0025	0.1888	0.0062	0.0403	0.0025	0.1154	0.0033	0.0211	0.0001	0.0240	0.0013	0.0377	0.0021	0.0217	0.0017	0.0271	0.0008	0.0175	0.0006	0.0436	0.0019	0.0134	0.0005	0.1172	0.0073	0.0195	0.0010
YR6	0.4418	0.0073	0.0571	0.0024	0.0571	0.0090	0.0388	0.0028	0.1681	0.0063	0.0196	0.0006	0.0470	0.0014	0.0469	0.0034	0.0242	0.0016	0.0345	0.0012	0.0155	0.0015	0.0456	0.0016	0.0152	0.0002	0.1413	0.0051	0.0179	0.0006
YR7	0.3382	0.0319	0.0506	0.0044	0.0506	0.0209	0.0418	0.0091	0.1138	0.0244	0.0178	0.0014	0.0212	0.0030	0.0276	0.0053	0.0163	0.0017	0.0264	0.0023	0.0117	0.0012	0.0375	0.0033	0.0089	0.0005	0.1258	0.0045	0.0154	0.0008
YR8	0.2821	0.0066	0.0310	0.0011	0.0310	0.0042	0.0274	0.0011	0.0635	0.0024	0.0048	0.0001	0.0119	0.0006	0.0162	0.0015	0.0123	0.0011	0.0209	0.0008	0.0089	0.0005	0.0313	0.0005	0.0073	0.0002	0.0895	0.0025	0.0116	0.0011
YR9	0.2982	0.0083	0.0403	0.0014	0.0403	0.0116	0.0288	0.0029	0.0826	0.0024	0.0162	0.0005	0.0157	0.0006	0.0187	0.0017	0.0136	0.0012	0.0194	0.0004	0.0097	0.0005	0.0288	0.0007	0.0084	0.0002	0.0925	0.0016	0.0130	0.0005
YR10	0.2727	0.0066	0.0263	0.0027	0.0263	0.0098	0.0181	0.0020	0.0653	0.0061	0.0100	0.0005	0.0183	0.0006	0.0185	0.0028	0.0095	0.0021	0.0191	0.0000	0.0075	0.0000	0.0302	0.0012	0.0082	0.0005	0.1021	0.0158	0.0119	0.0011
YR11	0.2965	0.0078	0.0359	0.0017	0.0359	0.0061	0.0307	0.0019	0.1078	0.0061	0.0168	0.0001	0.0215	0.0016	0.0211	0.0025	0.0099	0.0003	0.0225	0.0012	0.0087	0.0003	0.0349	0.0010	0.0087	0.0001	0.0921	0.0030	0.0130	0.0010
YR12	0.3535	0.0128	0.0586	0.0015	0.0586	0.0199	0.0433	0.0028	0.1668	0.0043	0.0116	0.0003	0.0230	0.0007	0.0425	0.0035	0.0103	0.0009	0.0275	0.0017	0.0093	0.0007	0.0311	0.0010	0.0077	0.0002	0.1044	0.0013	0.0113	0.0007
YR13	0.3259	0.0314	0.0311	0.0042	0.0311	0.0175	0.0242	0.0046	0.0640	0.0000	0.0121	0.0000	0.0265	0.0010	0.0232	0.0039	0.0085	0.0033	0.0208	0.0020	0.0092	0.0019	0.0391	0.0053	0.0079	0.0005	0.1104	0.0000	0.0124	0.0015
YR14	0.3709	0.0088	0.0472	0.0011	0.0472	0.0084	0.0379	0.0026	0.1132	0.0028	0.0146	0.0004	0.0201	0.0006	0.0316	0.0024	0.0101	0.0014	0.0258	0.0005	0.0096	0.0003	0.0426	0.0005	0.0064	0.0003	0.1404	0.0053	0.0109	0.0003
YR15	0.2745	0.0051	0.0274	0.0008	0.0274	0.0042	0.0198	0.0009	0.0782	0.0021	0.0088	0.0000	0.0204	0.0003	0.0227	0.0005	0.0088	0.0006	0.0226	0.0008	0.0084	0.0003	0.0321	0.0012	0.0057	0.0001	0.1025	0.0043	0.0091	0.0005
YR16	0.4208	0.0175	0.0503	0.0040	0.0503	0.0077	0.0382	0.0019	0.1352	0.0048	0.0157	0.0004	0.0336	0.0017	0.0357	0.0038	0.0077	0.0007	0.0292	0.0018	0.0094	0.0004	0.0418	0.0019	0.0087	0.0005	0.1222	0.0037	0.0107	0.0005
YR17	0.3051	0.0077	0.0281	0.0017	0.0281	0.0022	0.0166	0.0006	0.0756	0.0026	0.0108	0.0003	0.0216	0.0013	0.0234	0.0011	0.0072	0.0009	0.0212	0.0003	0.0061	0.0004	0.0299	0.0011	0.0056	0.0001	0.0969	0.0038	0.0086	0.0001
YR18	0.3191	0.0039	0.0259	0.0011	0.0259	0.0014	0.0204	0.0024	0.0727	0.0042	0.0098	0.0002	0.0135	0.0007	0.0205	0.0011	0.0064	0.0007	0.0225	0.0005	0.0063	0.0005	0.0346	0.0014	0.0056	0.0003	0.1150	0.0027	0.0085	0.0001
YR19	0.3512	0.0107	0.0417	0.0021	0.0417	0.0074	0.0292	0.0008	0.1069	0.0016	0.0160	0.0002	0.0186	0.0004	0.0317	0.0024	0.0075	0.0006	0.0224	0.0014	0.0084	0.0008	0.0397	0.0015	0.0076	0.0005	0.1221	0.0046	0.0089	0.0004
YR20	0.3347	0.0096	0.0358	0.0020	0.0358	0.0102	0.0229	0.0007	0.0894	0.0036	0.0082	0.0002	0.0103	0.0005	0.0249	0.0014	0.0061	0.0008	0.0228	0.0007	0.0082	0.0004	0.0435	0.0008	0.0077	0.0001	0.1283	0.0086	0.0136	0.0008
YR21	0.3301	0.0076	0.0425	0.0001	0.0425	0.0097	0.0302	0.0012	0.1051	0.0026	0.0151	0.0003	0.0173	0.0007	0.0223	0.0025	0.0058	0.0005	0.0271	0.0017	0.0088	0.0004	0.0340	0.0005	0.0066	0.0004	0.1131	0.0028	0.0090	0.0004
YR22	0.4861	0.0064	0.1032	0.0049	0.1032	0.0115	0.0954	0.0120	0.3061	0.0102	0.0386	0.0030	0.0336	0.0044	0.0693	0.0050	0.0132	0.0010	0.0385	0.0045	0.0139	0.0028	0.0530	0.0017	0.0072	0.0011	0.1268	0.0151	0.0104	0.0016

Table S6 Data related to the exposure and acute toxicity of nutrients and REEs, including information on transformed-normal distribution, calculated means, standard deviations, and results of the Kolmogorov-Smirnov test (K-S test).

Parameter	Ln-transformed exposure concentrations (μg/L)			Ln-transformed toxicity Concentrations (μg/L)		
	Mean	SD	K-S test	Mean	SD	K-S test
NH_4^+	5.39	0.72	0.088	16.03	1.10	0.19
NO_3^-	5.74	0.19	0.196	13.73	0.86	0.19
P	4.16	0.98	0.102	21.10	1.99	0.20
Y	-1.62	0.22	0.171	8.69	2.06	0.21
La	-2.56	0.45	0.124	9.41	1.50	0.20
Ce	-1.95	0.43	0.136	9.21	1.64	0.14
Pr	-4.10	0.59	0.182	8.91	1.55	0.27
Nd	-2.78	0.52	0.176	8.43	1.68	0.15
Sm	-4.24	0.64	0.153	8.71	1.24	0.21
Eu	-4.06	0.50	0.14	8.82	1.53	0.20
Gd	-3.92	0.52	0.156	8.35	1.17	0.12
Tb	-4.70	0.67	0.182	8.25	1.24	0.18
Dy	-3.68	0.34	0.201	8.32	1.12	0.16
Ho	-4.74	0.51	0.267	8.25	1.16	0.16
Er	-3.30	0.23	0.118	8.34	1.12	0.21
Tm	-4.93	0.53	0.297	8.30	1.23	0.18
Yb	-3.38	0.24	0.158	7.89	2.03	0.14
Lu	-4.22	0.46	0.205	8.30	1.90	0.20

References

Borgmann, U., Couillard, Y., Doyle, P., Dixon, D.G., 2005. Toxicity of sixty-three metals and metalloids to Hyalella azteca at two levels of water hardness. Environ. Toxicol. Chem. 24, 641–652.

Cui, J.A., 2011. Effects of REEs on Development of Zebrafish (Danio rerio). Vol. M.S. Qingdao University of Science & Technology. p. 61.

Dubé, M., Auclair, J., Hanana, H., Turcotte, P., Gagnon, C., Gagné, F., 2019. Gene expression changes and toxicity of selected rare earth elements in rainbow trout juveniles. Comp. Biochem. Physiol. C Toxicol. Pharmacol. 223, 88–95.

Gonzalez, V., Vignati, D.A.L., Leyval, C., Giamberini, L., 2014. Environmental fate and ecotoxicity of lanthanides: are they a uniform group beyond chemistry? Environ. Int. 71, 148–157.

Gu, Y.G., Gao, Y.P., Jordan, R.W., Jiang, S.J.,2025. Dynamics of metal immobilization in coastal mariculture sediments: Insights from DGT and DIFS modeling. Environ. Pollut. 383, 126798.

Rucki, M., Kejlova, K., Vlkova, K., Jirova, D., Dvorakova, M., Svobodova, L., Kandarova, H. Letasiova, S., Kolarova, H., Mannerstrom, M., Heinonen, T., 2020. Evaluation of toxicity profiles of rare earth elements salts (lanthanides). Rare Earths. 39, 225–232.

Su, D., Tai, P.D., Li, P.J., Ke, X., 2005. Toxic effects of lanthanides on Chlorella autotrophica. Chinese Journal of Ecology. 24, 382–384 (in Chinese with English abstract).

Tai, P.D., Zhao, Q., Su, D., Li, P.J., Stagnitti, F., 2010. Biological toxicity of lanthanide elements on algae. Chemosphere. 80, 1031–1035.

USEPA, 2001. Risk Assessment Guidance for Superfund: Volume III - Part A, Process for Conducting Probabilistic Risk Assessment. https://www.epa.gov/sites/production/files/2015-09/documents/rags3adt_complete.pdf.
